# Assessing “Dangerous Climate Change”: Required Reduction of Carbon Emissions to Protect Young People, Future Generations and Nature

**DOI:** 10.1371/journal.pone.0081648

**Published:** 2013-12-03

**Authors:** James Hansen, Pushker Kharecha, Makiko Sato, Valerie Masson-Delmotte, Frank Ackerman, David J. Beerling, Paul J. Hearty, Ove Hoegh-Guldberg, Shi-Ling Hsu, Camille Parmesan, Johan Rockstrom, Eelco J. Rohling, Jeffrey Sachs, Pete Smith, Konrad Steffen, Lise Van Susteren, Karina von Schuckmann, James C. Zachos

**Affiliations:** 1 Earth Institute, Columbia University, New York, New York, United States of America; 2 Goddard Institute for Space Studies, NASA, New York, New York, United States of America; 3 Institut Pierre Simon Laplace, Laboratoire des Sciences du Climat et de l’Environnement (CEA-CNRS-UVSQ), Gif-sur-Yvette, France; 4 Synapse Energy Economics, Cambridge, Massachusetts, United States of America; 5 Department of Animal and Plant Sciences, University of Sheffield, Sheffield, South Yorkshire, United Kingdom; 6 Department of Environmental Studies, University of North Carolina, Wilmington, North Carolina, United States of America; 7 Global Change Institute, University of Queensland, St. Lucia, Queensland, Australia; 8 College of Law, Florida State University, Tallahassee, Florida, United States of America; 9 Marine Institute, Plymouth University, Plymouth, Devon, United Kingdom; 10 Integrative Biology, University of Texas, Austin, Texas, United States of America; 11 Stockholm Resilience Center, Stockholm University, Stockholm, Sweden; 12 School of Ocean and Earth Science, University of Southampton, Southampton, Hampshire, United Kingdom; 13 Research School of Earth Sciences, Australian National University, Canberra, ACT, Australia; 14 University of Aberdeen, Aberdeen, Scotland, United Kingdom; 15 Swiss Federal Institute of Technology, Swiss Federal Research Institute WSL, Zurich, Switzerland; 16 Center for Health and the Global Environment, Advisory Board, Harvard School of Public Health, Boston, Massachusetts, United States of America; 17 L’Institut Francais de Recherche pour l’Exploitation de la Mer, Ifremer, Toulon, France; 18 Earth and Planetary Science, University of California, Santa Cruz, CA, United States of America; University of Oxford, United Kingdom

## Abstract

We assess climate impacts of global warming using ongoing observations and paleoclimate data. We use Earth’s measured energy imbalance, paleoclimate data, and simple representations of the global carbon cycle and temperature to define emission reductions needed to stabilize climate and avoid potentially disastrous impacts on today’s young people, future generations, and nature. A cumulative industrial-era limit of ∼500 GtC fossil fuel emissions and 100 GtC storage in the biosphere and soil would keep climate close to the Holocene range to which humanity and other species are adapted. Cumulative emissions of ∼1000 GtC, sometimes associated with 2°C global warming, would spur “slow” feedbacks and eventual warming of 3–4°C with disastrous consequences. Rapid emissions reduction is required to restore Earth’s energy balance and avoid ocean heat uptake that would practically guarantee irreversible effects. Continuation of high fossil fuel emissions, given current knowledge of the consequences, would be an act of extraordinary witting intergenerational injustice. Responsible policymaking requires a rising price on carbon emissions that would preclude emissions from most remaining coal and unconventional fossil fuels and phase down emissions from conventional fossil fuels.

## Introduction

Humans are now the main cause of changes of Earth’s atmospheric composition and thus the drive for future climate change [1]. The principal climate forcing, defined as an imposed change of planetary energy balance [1]–[2], is increasing carbon dioxide (CO_2_) from fossil fuel emissions, much of which will remain in the atmosphere for millennia [1], [3]. The climate response to this forcing and society’s response to climate change are complicated by the system’s inertia, mainly due to the ocean and the ice sheets on Greenland and Antarctica together with the long residence time of fossil fuel carbon in the climate system. The inertia causes climate to appear to respond slowly to this human-made forcing, but further long-lasting responses can be locked in.

**Figure 1:**
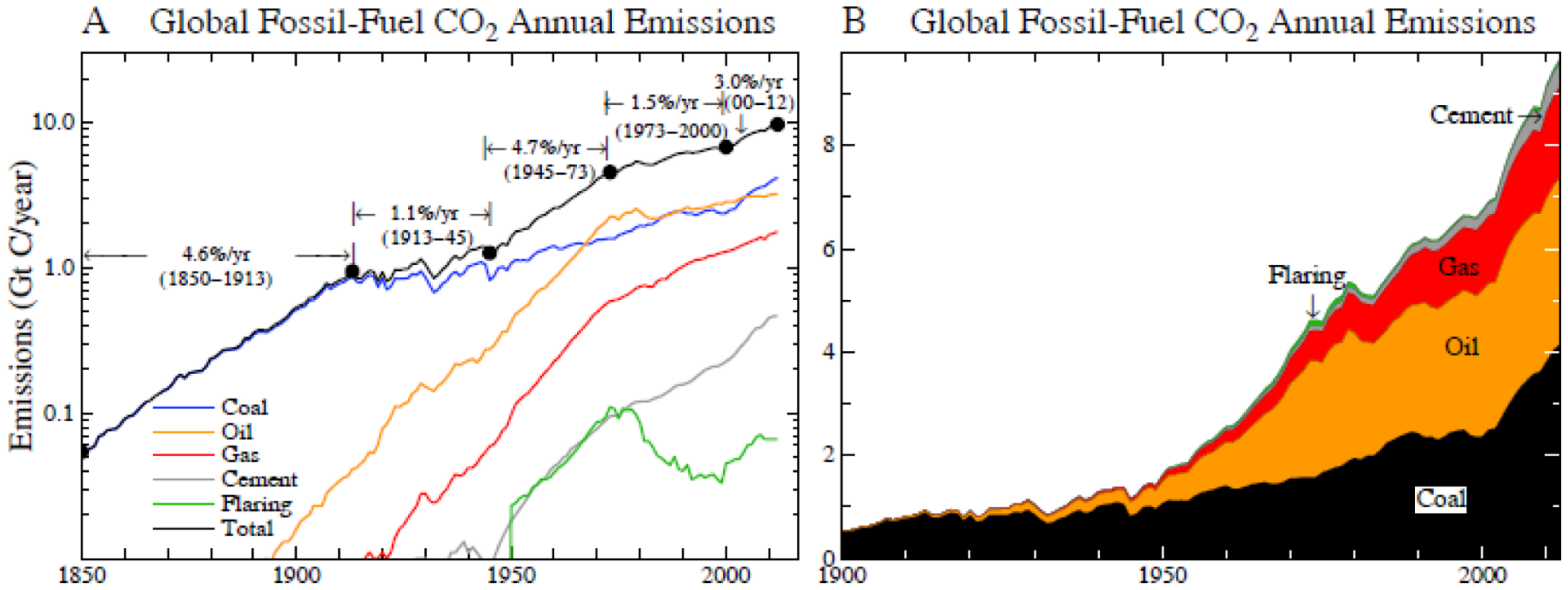
CO_2_ annual emissions from fossil fuel use and cement manufacture, based on data of British Petroleum [4] concatenated with data of Boden et al. [5]. (A) is log scale and (B) is linear.

More than 170 nations have agreed on the need to limit fossil fuel emissions to avoid dangerous human-made climate change, as formalized in the 1992 Framework Convention on Climate Change [6]. However, the stark reality is that global emissions have accelerated (Fig. 1) and new efforts are underway to massively expand fossil fuel extraction [7]–[9] by drilling to increasing ocean depths and into the Arctic, squeezing oil from tar sands and tar shale, hydro-fracking to expand extraction of natural gas, developing exploitation of methane hydrates, and mining of coal via mountaintop removal and mechanized long-wall mining. The growth rate of fossil fuel emissions increased from 1.5%/year during 1980–2000 to 3%/year in 2000–2012, mainly because of increased coal use [4]–[5].

The Framework Convention [6] does not define a dangerous level for global warming or an emissions limit for fossil fuels. The European Union in 1996 proposed to limit global warming to 2°C relative to pre-industrial times [10], based partly on evidence that many ecosystems are at risk with larger climate change. The 2°C target was reaffirmed in the 2009 “Copenhagen Accord” emerging from the 15th Conference of the Parties of the Framework Convention [11], with specific language “We agree that deep cuts in global emissions are required according to science, as documented in the IPCC Fourth Assessment Report with a view to reduce global emissions so as to hold the increase in global temperature below 2 degrees Celsius…”.

A global warming target is converted to a fossil fuel emissions target with the help of global climate-carbon-cycle models, which reveal that eventual warming depends on cumulative carbon emissions, not on the temporal history of emissions [12]. The emission limit depends on climate sensitivity, but central estimates [12]–[13], including those in the upcoming Fifth Assessment of the Intergovernmental Panel on Climate Change [14], are that a 2°C global warming limit implies a cumulative carbon emissions limit of the order of 1000 GtC. In comparing carbon emissions, note that some authors emphasize the sum of fossil fuel and deforestation carbon. We bookkeep fossil fuel and deforestation carbon separately, because the larger fossil fuel term is known more accurately and this carbon stays in the climate system for hundreds of thousands of years. Thus fossil fuel carbon is the crucial human input that must be limited. Deforestation carbon is more uncertain and potentially can be offset on the century time scale by storage in the biosphere, including the soil, via reforestation and improved agricultural and forestry practices.

**Figure 2:**
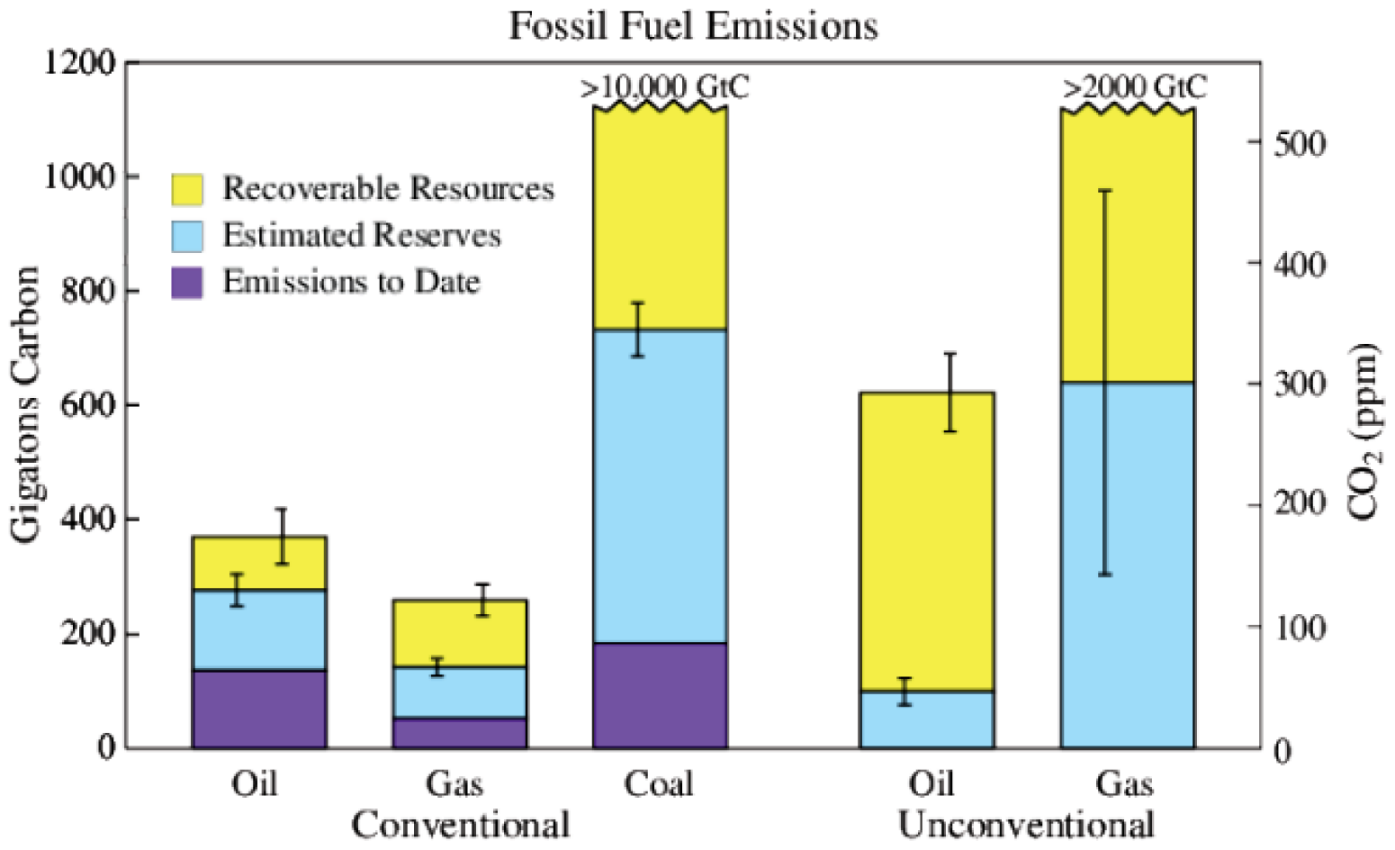
Fossil fuel CO_2_ emissions and carbon content (1 ppm atmospheric CO_2_ ∼ 2.12 GtC). Estimates of reserves (profitable to extract at current prices) and resources (potentially recoverable with advanced technology and/or at higher prices) are the mean of estimates of Energy Information Administration (EIA) [7], German Advisory Council (GAC) [8], and Global Energy Assessment (GEA) [9]. GEA [9] suggests the possibility of >15,000 GtC unconventional gas. Error estimates (vertical lines) are from GEA and probably underestimate the total uncertainty. We convert energy content to carbon content using emission factors of Table 4.2 of [15] for coal, gas and conventional oil, and, also following [15], emission factor of unconventional oil is approximated as being the same as for coal. Total emissions through 2012, including gas flaring and cement manufacture, are 384 GtC; fossil fuel emissions alone are ∼370 GtC.

There are sufficient fossil fuel resources to readily supply 1000 GtC, as fossil fuel emissions to date (370 GtC) are only a small fraction of potential emissions from known reserves and potentially recoverable resources (Fig. 2). Although there are uncertainties in reserves and resources, ongoing fossil fuel subsidies and continuing technological advances ensure that more and more of these fuels will be economically recoverable. As we will show, Earth’s paleoclimate record makes it clear that the CO_2_ produced by burning all or most of these fossil fuels would lead to a very different planet than the one that humanity knows.

Our evaluation of a fossil fuel emissions limit is not based on climate models but rather on observational evidence of global climate change as a function of global temperature and on the fact that climate stabilization requires long-term planetary energy balance. We use measured global temperature and Earth’s measured energy imbalance to determine the atmospheric CO_2_ level required to stabilize climate at today’s global temperature, which is near the upper end of the global temperature range in the current interglacial period (the Holocene). We then examine climate impacts during the past few decades of global warming and in paleoclimate records including the Eemian period, concluding that there are already clear indications of undesirable impacts at the current level of warming and that 2°C warming would have major deleterious consequences. We use simple representations of the carbon cycle and global temperature, consistent with observations, to simulate transient global temperature and assess carbon emission scenarios that could keep global climate near the Holocene range. Finally, we discuss likely over-shooting of target emissions, the potential for carbon extraction from the atmosphere, and implications for energy and economic policies, as well as intergenerational justice.

## Global Temperature and Earth’s Energy Balance

Global temperature and Earth’s energy imbalance provide our most useful measuring sticks for quantifying global climate change and the changes of global climate forcings that would be required to stabilize global climate. Thus we must first quantify knowledge of these quantities.

### Temperature

**Figure 3:**
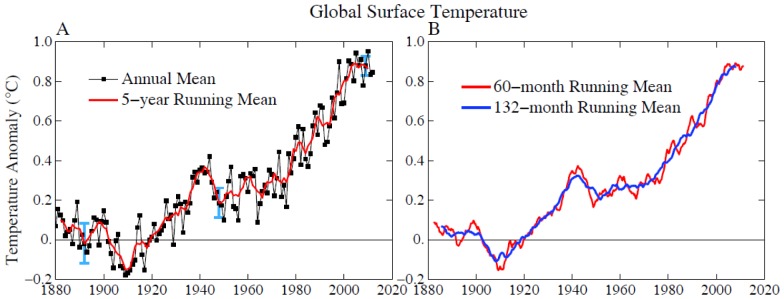
Global surface temperature relative to 1880–1920 mean. B shows the 5 and 11 year means. Figures are updates of [16] using data through August 2013.

Temperature change in the past century (Fig. 3; update of figures in [16]) includes unforced variability and forced climate change. The long-term global warming trend is predominantly a forced climate change caused by increased human-made atmospheric gases, mainly CO_2_ [1]. Increase of “greenhouse” gases such as CO_2_ has little effect on incoming sunlight but makes the atmosphere more opaque at infrared wavelengths, causing infrared (heat) radiation to space to emerge from higher, colder levels, which thus reduces infrared radiation to space. The resulting planetary energy imbalance, absorbed solar energy exceeding heat emitted to space, causes Earth to warm. Observations, discussed below, confirm that Earth is now substantially out of energy balance, so the long-term warming will continue.

Global temperature appears to have leveled off since 1998 (Fig. 3a). That plateau is partly an illusion due to the 1998 global temperature spike caused by the El Niño of the century that year. The 11-year (132-month) running mean temperature (Fig. 3b) shows only a moderate decline of the warming rate. The 11-year averaging period minimizes the effect of variability due to the 10–12 year periodicity of solar irradiance as well as irregular El Niño/La Niña warming/cooling in the tropical Pacific Ocean. The current solar cycle has weaker irradiance than the several prior solar cycles, but the decreased irradiance can only partially account for the decreased warming rate [17]. Variability of the El Niño/La Niña cycle, described as a Pacific Decadal Oscillation, largely accounts for the temporary decrease of warming [18], as we discuss further below in conjunction with global temperature simulations.

Assessments of dangerous climate change have focused on estimating a permissible level of global warming. The Intergovernmental Panel on Climate Change [1], [19] summarized broad-based assessments with a “burning embers” diagram, which indicated that major problems begin with global warming of 2–3°C. A probabilistic analysis [20], still partly subjective, found a median “dangerous” threshold of 2.8°C, with 95% confidence that the dangerous threshold was 1.5°C or higher. These assessments were relative to global temperature in year 1990, so add 0.6°C to these values to obtain the warming relative to 1880–1920, which is the base period we use in this paper for preindustrial time. The conclusion that humanity could tolerate global warming up to a few degrees Celsius meshed with common sense. After all, people readily tolerate much larger regional and seasonal climate variations.

The fallacy of this logic emerged recently as numerous impacts of ongoing global warming emerged and as paleoclimate implications for climate sensitivity became apparent. Arctic sea ice end-of-summer minimum area, although variable from year to year, has plummeted by more than a third in the past few decades, at a faster rate than in most models [21], with the sea ice thickness declining a factor of four faster than simulated in IPCC climate models [22]. The Greenland and Antarctic ice sheets began to shed ice at a rate, now several hundred cubic kilometers per year, which is continuing to accelerate [23]–[25]. Mountain glaciers are receding rapidly all around the world [26]–[29] with effects on seasonal freshwater availability of major rivers [30]–[32]. The hot dry subtropical climate belts have expanded as the troposphere has warmed and the stratosphere cooled [33]–[36], contributing to increases in the area and intensity of drought [37] and wildfires [38]. The abundance of reef-building corals is decreasing at a rate of 0.5–2%/year, at least in part due to ocean warming and possibly ocean acidification caused by rising dissolved CO_2_ [39]–[41]. More than half of all wild species have shown significant changes in where they live and in the timing of major life events [42]–[44]. Mega-heatwaves, such as those in Europe in 2003, the Moscow area in 2010, Texas and Oklahoma in 2011, Greenland in 2012, and Australia in 2013 have become more widespread with the increase demonstrably linked to global warming [45]–[47].

These growing climate impacts, many more rapid than anticipated and occurring while global warming is less than 1°C, imply that society should reassess what constitutes a “dangerous level” of global warming. Earth’s paleoclimate history provides a valuable tool for that purpose.

### Paleoclimate Temperature

Major progress in quantitative understanding of climate change has occurred recently by use of the combination of data from high resolution ice cores covering time scales of order several hundred thousand years [48]–[49] and ocean cores for time scales of order one hundred million years [50]. Quantitative insights on global temperature sensitivity to external forcings [51]–[52] and sea level sensitivity to global temperature [52]–[53] are crucial to our analyses. Paleoclimate data also provide quantitative information about how nominally slow feedback processes amplify climate sensitivity [51]–[52], [54]–[56], which also is important to our analyses.

**Figure 4:**
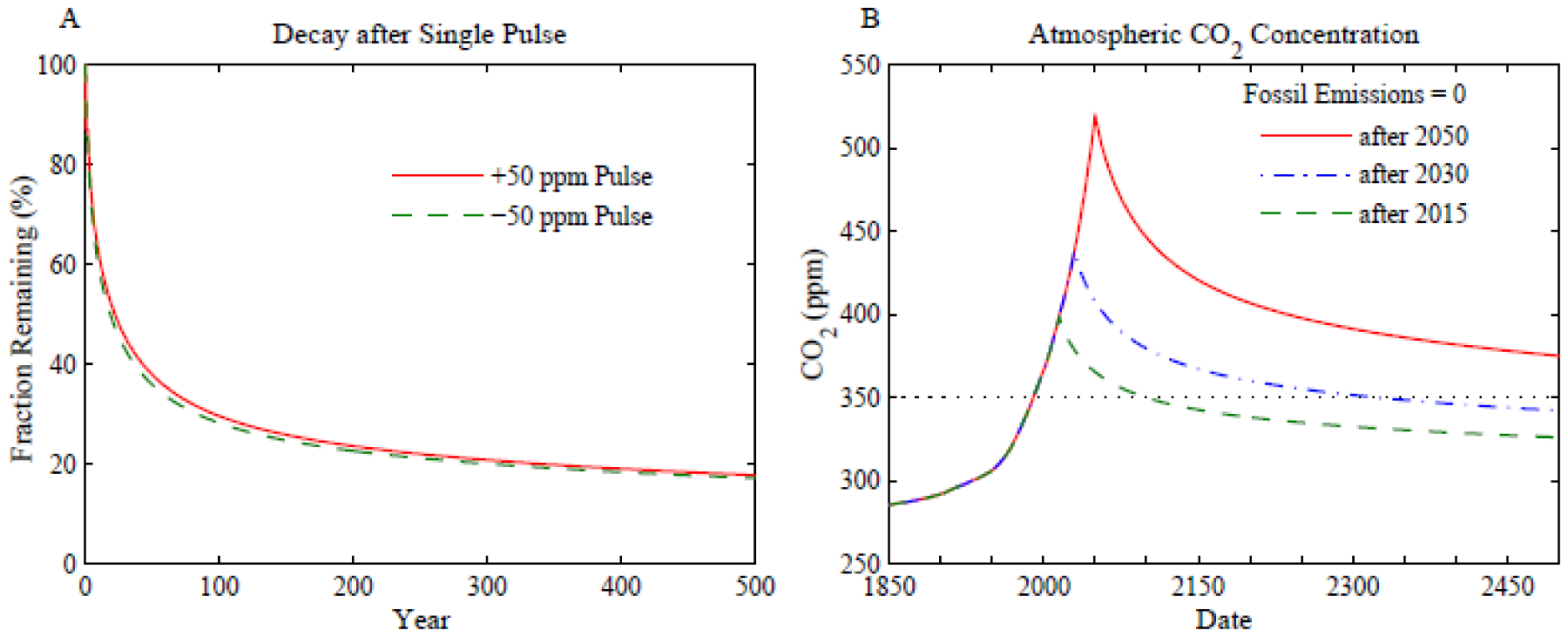
Decay of atmospheric CO_2_ perturbations. (A) Instantaneous injection or extraction of CO_2_ with initial conditions at equilibrium. (B) Fossil fuel emissions terminate at the end of 2015, 2030, or 2050 and land use emissions terminate after 2015 in all three cases, i.e., thereafter there is no net deforestation.

Earth’s surface temperature prior to instrumental measurements is estimated via proxy data. We will refer to the surface temperature record in Fig. 4 of a recent paper [52]. Global mean temperature during the Eemian interglacial period (120,000 years ago) is constrained to be 2°C warmer than our pre-industrial (1880–1920) level based on several studies of Eemian climate [52]. The concatenation of modern and instrumental records [52] is based on an estimate that global temperature in the first decade of the 21st century (+0.8°C relative to 1880–1920) exceeded the Holocene mean by 0.25±0.25°C. That estimate was based in part on the fact that sea level is now rising 3.2 mm/yr (3.2 m/millennium) [57], an order of magnitude faster than the rate during the prior several thousand years, with rapid change of ice sheet mass balance over the past few decades [23] and Greenland and Antarctica now losing mass at accelerating rates [23]–[24]. This concatenation, which has global temperature 13.9°C in the base period 1951–1980, has the first decade of the 21st century slightly (∼0.1°C) warmer than the early Holocene maximum. A recent reconstruction from proxy temperature data [55] concluded that global temperature declined about 0.7°C between the Holocene maximum and a pre-industrial minimum before recent warming brought temperature back near the Holocene maximum, which is consistent with our analysis.

Climate oscillations evident in Fig. 4 of Hansen et al. [52] were instigated by perturbations of Earth’s orbit and spin axis tilt relative to the orbital plane, which alter the geographical and seasonal distribution of sunlight on Earth [58]. These forcings change slowly, with periods between 20,000 and 400,000 years, and thus climate is able to stay in quasi-equilibrium with these forcings. Slow insolation changes initiated the climate oscillations, but the mechanisms that caused the climate changes to be so large were two powerful amplifying feedbacks: the planet’s surface albedo (its reflectivity, literally its whiteness) and atmospheric CO_2_ amount. As the planet warms, ice and snow melt, causing the surface to be darker, absorb more sunlight and warm further. As the ocean and soil become warmer they release CO_2_ and other greenhouse gases, causing further warming. Together with fast feedbacks processes, via changes of water vapor, clouds, and the vertical temperature profile, these slow amplifying feedbacks were responsible for almost the entire glacial-to-interglacial temperature change [59]–[62].

The albedo and CO_2_ feedbacks amplified weak orbital forcings, the feedbacks necessarily changing slowly over millennia, at the pace of orbital changes. Today, however, CO_2_ is under the control of humans as fossil fuel emissions overwhelm natural changes. Atmospheric CO_2_ has increased rapidly to a level not seen for at least 3 million years [56], [63]. Global warming induced by increasing CO_2_ will cause ice to melt and hence sea level to rise as the global volume of ice moves toward the quasi-equilibrium amount that exists for a given global temperature [53]. As ice melts and ice area decreases, the albedo feedback will amplify global warming.

Earth, because of the climate system’s inertia, has not yet fully responded to human-made changes of atmospheric composition. The ocean’s thermal inertia, which delays some global warming for decades and even centuries, is accounted for in global climate models and its effect is confirmed via measurements of Earth’s energy balance (see next section). In addition there are slow climate feedbacks, such as changes of ice sheet size, that occur mainly over centuries and millennia. Slow feedbacks have little effect on the immediate planetary energy balance, instead coming into play in response to temperature change. The slow feedbacks are difficult to model, but paleoclimate data and observations of ongoing changes help provide quantification.

### Earth’s Energy Imbalance

At a time of climate stability, Earth radiates as much energy to space as it absorbs from sunlight. Today Earth is out of balance because increasing atmospheric gases such as CO_2_ reduce Earth’s heat radiation to space, thus causing an energy imbalance, as there is less energy going out than coming in. This imbalance causes Earth to warm and move back toward energy balance. The warming and restoration of energy balance take time, however, because of Earth’s thermal inertia, which is due mainly to the global ocean.

Earth warmed about 0.8°C in the past century. That warming increased Earth’s radiation to space, thus reducing Earth’s energy imbalance. The remaining energy imbalance helps us assess how much additional warming is still “in the pipeline”. Of course increasing CO_2_ is only one of the factors affecting Earth’s energy balance, even though it is the largest climate forcing. Other forcings include changes of aerosols, solar irradiance, and Earth’s surface albedo.

Determination of the state of Earth’s climate therefore requires measuring the energy imbalance. This is a challenge, because the imbalance is expected to be only about 1 W/m^2^ or less, so accuracy approaching 0.1 W/m^2^ is needed. The most promising approach is to measure the rate of changing heat content of the ocean, atmosphere, land, and ice [64]. Measurement of ocean heat content is the most critical observation, as nearly 90 percent of the energy surplus is stored in the ocean [64]–[65].

### Observed Energy Imbalance

Nations of the world have launched a cooperative program to measure changing ocean heat content, distributing more than 3000 Argo floats around the world ocean, with each float repeatedly diving to a depth of 2 km and back [66]. Ocean coverage by floats reached 90% by 2005 [66], with the gaps mainly in sea ice regions, yielding the potential for an accurate energy balance assessment, provided that several systematic measurement biases exposed in the past decade are minimized [67]–[69].

Argo data reveal that in 2005–2010 the ocean’s upper 2000 m gained heat at a rate equal to 0.41 W/m^2^ averaged over Earth’s surface [70]. Smaller contributions to planetary energy imbalance are from heat gain by the deeper ocean (+0.10 W/m^2^), energy used in net melting of ice (+0.05 W/m^2^), and energy taken up by warming continents (+0.02 W/m^2^). Data sources for these estimates and uncertainties are provided elsewhere [64]. The resulting net planetary energy imbalance for the six years 2005–2010 is +0.58±0.15 W/m^2^.

The positive energy imbalance in 2005–2010 confirms that the effect of solar variability on climate is much less than the effect of human-made greenhouse gases. If the sun were the dominant forcing, the planet would have a negative energy balance in 2005–2010, when solar irradiance was at its lowest level in the period of accurate data, i.e., since the 1970s [64], [71]. Even though much of the greenhouse gas forcing has been expended in causing observed 0.8°C global warming, the residual positive forcing overwhelms the negative solar forcing. The full amplitude of solar cycle forcing is about 0.25 W/m^2^ [64], [71], but the reduction of solar forcing due to the present weak solar cycle is about half that magnitude as we illustrate below, so the energy imbalance measured during solar minimum (0.58 W/m^2^) suggests an average imbalance over the solar cycle of about 0.7 W/m^2^.

Earth’s measured energy imbalance has been used to infer the climate forcing by aerosols, with two independent analyses yielding a forcing in the past decade of about −1.5 W/m^2^ [64], [72], including the direct aerosol forcing and indirect effects via induced cloud changes. Given this large (negative) aerosol forcing, precise monitoring of changing aerosols is needed [73]. Public reaction to increasingly bad air quality in developing regions [74] may lead to future aerosol reductions, at least on a regional basis. Increase of Earth’s energy imbalance from reduction of particulate air pollution, which is needed for the sake of human health, can be minimized via an emphasis on reducing absorbing black soot [75], but the potential to constrain the net increase of climate forcing by focusing on black soot is limited [76].

### Energy Imbalance Implications for CO_2_ Target

Earth’s energy imbalance is the most vital number characterizing the state of Earth’s climate. It informs us about the global temperature change “in the pipeline” without further change of climate forcings and it defines how much greenhouse gases must be reduced to restore Earth’s energy balance, which, at least to a good approximation, must be the requirement for stabilizing global climate. The measured energy imbalance accounts for all natural and human-made climate forcings, including changes of atmospheric aerosols and Earth’s surface albedo.

If Earth’s mean energy imbalance today is +0.5 W/m^2^, CO_2_ must be reduced from the current level of 395 ppm (global-mean annual-mean in mid-2013) to about 360 ppm to increase Earth’s heat radiation to space by 0.5 W/m^2^ and restore energy balance. If Earth’s energy imbalance is 0.75 W/m^2^, CO_2_ must be reduced to about 345 ppm to restore energy balance [64], [75].

The measured energy imbalance indicates that an initial CO_2_ target “<350 ppm” would be appropriate, if the aim is to stabilize climate without further global warming. That target is consistent with an earlier analysis [54]. Additional support for that target is provided by our analyses of ongoing climate change and paleoclimate, in later parts of our paper. Specification now of a CO_2_ target more precise than <350 ppm is difficult and unnecessary, because of uncertain future changes of forcings including other gases, aerosols and surface albedo. More precise assessments will become available during the time that it takes to turn around CO_2_ growth and approach the initial 350 ppm target.

Below we find the decreasing emissions scenario that would achieve the 350 ppm target within the present century. Specifically, we want to know the annual percentage rate at which emissions must be reduced to reach this target, and the dependence of this rate upon the date at which reductions are initiated. This approach is complementary to the approach of estimating cumulative emissions allowed to achieve a given limit on global warming [12].

If the only human-made climate forcing were changes of atmospheric CO_2_, the appropriate CO_2_ target might be close to the pre-industrial CO_2_ amount [53]. However, there are other human forcings, including aerosols, the effect of aerosols on clouds, non-CO_2_ greenhouse gases, and changes of surface albedo that will not disappear even if fossil fuel burning is phased out. Aerosol forcings are substantially a result of fossil fuel burning [1], [76], but the net aerosol forcing is a sensitive function of various aerosol sources [76]. The indirect aerosol effect on clouds is non-linear [1], [76] such that it has been suggested that even the modest aerosol amounts added by pre-industrial humans to an otherwise pristine atmosphere may have caused a significant climate forcing [59]. Thus continued precise monitoring of Earth’s radiation imbalance is probably the best way to assess and adjust the appropriate CO_2_ target.

Ironically, future reductions of particulate air pollution may exacerbate global warming by reducing the cooling effect of reflective aerosols. However, a concerted effort to reduce non-CO_2_ forcings by methane, tropospheric ozone, other trace gases, and black soot might counteract the warming from a decline in reflective aerosols [54], [75]. Our calculations below of future global temperature assume such compensation, as a first approximation. To the extent that goal is not achieved, adjustments must be made in the CO_2_ target or future warming may exceed calculated values.

## Climate Impacts

Determination of the dangerous level of global warming inherently is partly subjective, but we must be as quantitative as possible. Early estimates for dangerous global warming based on the “burning embers” approach [1], [19]–[20] have been recognized as probably being too conservative [77]. A target of limiting warming to 2°C has been widely adopted, as discussed above. We suspect, however, that this may be a case of inching toward a better answer. If our suspicion is correct, then that gradual approach is itself very dangerous, because of the climate system's inertia. It will become exceedingly difficult to keep warming below a target smaller than 2°C, if high emissions continue much longer.

We consider several important climate impacts and use evidence from current observations to assess the effect of 0.8°C warming and paleoclimate data for the effect of larger warming, especially the Eemian period, which had global mean temperature about +2°C relative to pre-industrial time. Impacts of special interest are sea level rise and species extermination, because they are practically irreversible, and others important to humankind.

### Sea Level

The prior interglacial period, the Eemian, was at most ∼2°C warmer than 1880–1920 (Fig. 3). Sea level reached heights several meters above today’s level [78]–[80], probably with instances of sea level change of the order of 1 m/century [81]–[83]. Geologic shoreline evidence has been interpreted as indicating a rapid sea level rise of a few meters late in the Eemian to a peak about 9 meters above present, suggesting the possibility that a critical stability threshold was crossed that caused polar ice sheet collapse [84]–[85], although there remains debate within the research community about this specific history and interpretation. The large Eemian sea level excursions imply that substantial ice sheet melting occurred when the world was little warmer than today.

During the early Pliocene, which was only ∼3°C warmer than the Holocene, sea level attained heights as much as 15–25 meters higher than today [53], [86]–[89]. Such sea level rise suggests that parts of East Antarctica must be vulnerable to eventual melting with global temperature increase of a few degrees Celsius. Indeed, satellite gravity data and radar altimetry reveal that the Totten Glacier of East Antarctica, which fronts a large ice mass grounded below sea level, is now losing mass [90].

Greenland ice core data suggest that the Greenland ice sheet response to Eemian warmth was limited [91], but the fifth IPCC assessment [14] concludes that Greenland very likely contributed between 1.4 and 4.3 m to the higher sea level of the Eemian. The West Antarctic ice sheet is probably more susceptible to rapid change, because much of it rests on bedrock well below sea level [92]–[93]. Thus the entire 3–4 meters of global sea level contained in that ice sheet may be vulnerable to rapid disintegration, although arguments for stability of even this marine ice sheet have been made [94]. However, Earth’s history reveals sea level changes of as much as a few meters per century, even though the natural climate forcings changed much more slowly than the present human-made forcing.

Expected human-caused sea level rise is controversial in part because predictions focus on sea level at a specific time, 2100. Sea level on a given date is inherently difficult to predict, as it depends on how rapidly non-linear ice sheet disintegration begins. Focus on a single date also encourages people to take the estimated result as an indication of what humanity faces, thus failing to emphasize that the likely rate of sea level rise immediately after 2100 will be much larger than within the 21^st^ century, especially if CO_2_ emissions continue to increase.

Recent estimates of sea level rise by 2100 have been of the order of 1 m [95]–[96], which is higher than earlier assessments [26], but these estimates still in part assume linear relations between warming and sea level rise. It has been argued [97]–[98] that continued business-as-usual CO_2_ emissions are likely to spur a nonlinear response with multi-meter sea level rise this century. Greenland and Antarctica have been losing mass at rapidly increasing rates during the period of accurate satellite data [23]; the data are suggestive of exponential increase, but the records are too short to be conclusive. The area on Greenland with summer melt has increased markedly, with 97% of Greenland experiencing melt in 2012 [99].

The important point is that the uncertainty is not about whether continued rapid CO_2_ emissions would cause large sea level rise, submerging global coastlines – it is about how soon the large changes would begin. The carbon from fossil fuel burning will remain in and affect the climate system for many millennia, ensuring that over time sea level rise of many meters will occur – tens of meters if most of the fossil fuels are burned [53]. That order of sea level rise would result in the loss of hundreds of historical coastal cities worldwide with incalculable economic consequences, create hundreds of millions of global warming refugees from highly-populated low-lying areas, and thus likely cause major international conflicts.

### Shifting Climate Zones

Theory and climate models indicate that the tropical overturning (Hadley) atmospheric circulation expands poleward with global warming [33]. There is evidence in satellite and radiosonde data and in observational data for poleward expansion of the tropical circulation by as much as a few degrees of latitude since the 1970s [34]–[35], but natural variability may have contributed to that expansion [36]. Change in the overturning circulation likely contributes to expansion of subtropical conditions and increased aridity in the southern United States [30], [100], the Mediterranean region, South America, southern Africa, Madagascar, and southern Australia. Increased aridity and temperature contribute to increased forest fires that burn hotter and are more destructive [38].

Despite large year-to-year variability of temperature, decadal averages reveal isotherms (lines of a given average temperature) moving poleward at a typical rate of the order of 100 km/decade in the past three decades [101], although the range shifts for specific species follow more complex patterns [102]. This rapid shifting of climate zones far exceeds natural rates of change. Movement has been in the same direction (poleward, and upward in elevation) since about 1975. Wild species have responded to climate change, with three-quarters of marine species shifting their ranges poleward as much as 1000 km [44], [103] and more than half of terrestrial species shifting ranges poleward as much as 600 km and upward as much as 400 m [104].

Humans may adapt to shifting climate zones better than many species. However, political borders can interfere with human migration, and indigenous ways of life already have been adversely affected [26]. Impacts are apparent in the Arctic, with melting tundra, reduced sea ice, and increased shoreline erosion. Effects of shifting climate zones also may be important for indigenous Americans who possess specific designated land areas, as well as other cultures with long-standing traditions in South America, Africa, Asia and Australia.

### Human Extermination of Species

Biodiversity is affected by many agents including overharvesting, introduction of exotic species, land use changes, nitrogen fertilization, and direct effects of increased atmospheric CO_2_ on plant ecophysiology [43]. However, an overriding role of climate change is exposed by diverse effects of rapid warming on animals, plants, and insects in the past three decades.

A sudden widespread decline of frogs, with extinction of entire mountain-restricted species attributed to global warming [105]–[106], provided a dramatic awakening. There are multiple causes of the detailed processes involved in global amphibian declines and extinctions [107]–[108], but global warming is a key contributor and portends a planetary-scale mass extinction in the making unless action is taken to stabilize climate while also fighting biodiversity’s other threats [109].

Mountain-restricted and polar-restricted species are particularly vulnerable. As isotherms move up the mountainside and poleward, so does the climate zone in which a given species can survive. If global warming continues unabated, many of these species will be effectively pushed off the planet. There are already reductions in the population and health of Arctic species in the southern parts of the Arctic, Antarctic species in the northern parts of the Antarctic, and alpine species worldwide [43].

**Figure 5:**
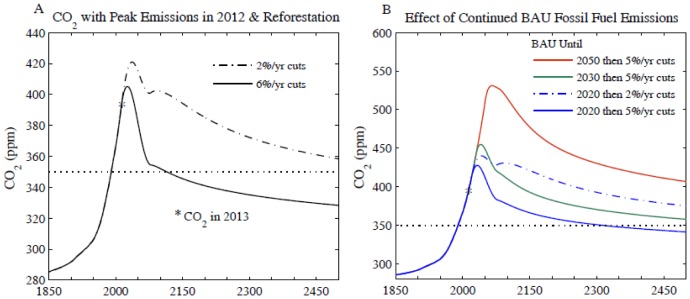
Atmospheric CO_2_ if fossil fuel emissions reduced. (A) 6% or 2% annual cut begins in 2013 and 100 GtC reforestation drawdown occurs in 2031–2080, (B) effect of delaying onset of emission reduction.

A critical factor for survival of some Arctic species is retention of all-year sea ice. Continued growth of fossil fuel emissions will cause loss of all Arctic summer sea ice within several decades. In contrast, the scenario in Fig. 5A, with global warming peaking just over 1°C and then declining slowly, should allow summer sea ice to survive and then gradually increase to levels representative of recent decades.

The threat to species survival is not limited to mountain and polar species. Plant and animal distributions reflect the regional climates to which they are adapted. Although species attempt to migrate in response to climate change, their paths may be blocked by human-constructed obstacles or natural barriers such as coast lines and mountain ranges. As the shift of climate zones [110] becomes comparable to the range of some species, less mobile species can be driven to extinction. Because of extensive species interdependencies, this can lead to mass extinctions.

Rising sea level poses a threat to a large number of uniquely evolved endemic fauna living on islands in marine-dominated ecosystems, with those living on low lying islands being especially vulnerable. Evolutionary history on Bermuda offers numerous examples of the direct and indirect impact of changing sea level on evolutionary processes [111]–[112], with a number of taxa being extirpated due to habitat changes, greater competition, and island inundation [113]. Similarly, on Aldahabra Island in the Indian Ocean, land tortoises were exterminated during sea level high stands [114]. Vulnerabilities would be magnified by the speed of human-made climate change and the potentially large sea level rise [115].

IPCC [26] reviewed studies relevant to estimating eventual extinctions. They estimate that if global warming exceeds 1.6°C above preindustrial, 9–31 percent of species will be committed to extinction. With global warming of 2.9°C, an estimated 21–52 percent of species will be committed to extinction. A comprehensive study of biodiversity indicators over the past decade [116] reveals that, despite some local success in increasing extent of protected areas, overall indicators of pressures on biodiversity including that due to climate change are continuing to increase and indicators of the state of biodiversity are continuing to decline.

Mass extinctions occurred several times in Earth’s history [117]–[118], often in conjunction with rapid climate change. New species evolved over millions of years, but those time scales are almost beyond human comprehension. If we drive many species to extinction we will leave a more desolate, monotonous planet for our children, grandchildren, and more generations than we can imagine. We will also undermine ecosystem functions (e.g., pollination which is critical for food production) and ecosystem resilience (when losing keystone species in food chains), as well as reduce functional diversity (critical for the ability of ecosystems to respond to shocks and stress) and genetic diversity that plays an important role for development of new medicines, materials, and sources of energy.

### Coral Reef Ecosystems

Coral reefs are the most biologically diverse marine ecosystem, often described as the rainforests of the ocean. Over a million species, most not yet described [119], are estimated to populate coral reef ecosystems generating crucial ecosystem services for at least 500 million people in tropical coastal areas. These ecosystems are highly vulnerable to the combined effects of ocean acidification and warming.

Acidification arises as the ocean absorbs CO_2_, producing carbonic acid [120], thus making the ocean more corrosive to the calcium carbonate shells (exoskeletons) of many marine organisms. Geochemical records show that ocean pH is already outside its range of the past several million years [121]–[122]. Warming causes coral bleaching, as overheated coral expel symbiotic algae and become vulnerable to disease and mortality [123]. Coral bleaching and slowing of coral calcification already are causing mass mortalities, increased coral disease, and reduced reef carbonate accretion, thus disrupting coral reef ecosystem health [40], [124].

Local human-made stresses add to the global warming and acidification effects, all of these driving a contraction of 1–2% per year in the abundance of reef-building corals [39]. Loss of the three-dimensional coral reef frameworks has consequences for all the species that depend on them. Loss of these frameworks also has consequences for the important roles that coral reefs play in supporting fisheries and protecting coastlines from wave stress. Consequences of lost coral reefs can be economically devastating for many nations, especially in combination with other impacts such as sea level rise and intensification of storms.

### Climate Extremes

Changes in the frequency and magnitude of climate extremes, of both moisture and temperature, are affected by climate trends as well as changing variability. Extremes of the hydrologic cycle are expected to intensify in a warmer world. A warmer atmosphere holds more moisture, so precipitation can be heavier and cause more extreme flooding. Higher temperatures, on the other hand, increase evaporation and can intensify droughts when they occur, as can expansion of the subtropics, as discussed above. Global models for the 21st century find an increased variability of precipitation minus evaporation [P-E] in most of the world, especially near the equator and at high latitudes [125]. Some models also show an intensification of droughts in the Sahel, driven by increasing greenhouse gases [126].

Observations of ocean salinity patterns for the past 50 years reveal an intensification of [P-E] patterns as predicted by models, but at an even faster rate. Precipitation observations over land show the expected general increase of precipitation poleward of the subtropics and decrease at lower latitudes [1], [26]. An increase of intense precipitation events has been found on much of the world’s land area [127]–[129]. Evidence for widespread drought intensification is less clear and inherently difficult to confirm with available data because of the increase of time-integrated precipitation at most locations other than the subtropics. Data analyses have found an increase of drought intensity at many locations [130]–[131] The magnitude of change depends on the drought index employed [132], but soil moisture provides a good means to separate the effect of shifting seasonal precipitation and confirms an overall drought intensification [37].

Global warming of ∼0.6°C since the 1970s (Fig. 3) has already caused a notable increase in the occurrence of extreme summer heat [46]. The likelihood of occurrence or the fractional area covered by 3-standard-deviation hot anomalies, relative to a base period (1951–1980) that was still within the range of Holocene climate, has increased by more than a factor of ten. Large areas around Moscow, the Mediterranean region, the United States and Australia have experienced such extreme anomalies in the past three years. Heat waves lasting for weeks have a devastating impact on human health: the European heat wave of summer 2003 caused over 70,000 excess deaths [133]. This heat record for Europe was surpassed already in 2010 [134]. The number of extreme heat waves has increased several-fold due to global warming [45]–[46], [135] and will increase further if temperatures continue to rise.

### Human Health

Impacts of climate change cause widespread harm to human health, with children often suffering the most. Food shortages, polluted air, contaminated or scarce supplies of water, an expanding area of vectors causing infectious diseases, and more intensely allergenic plants are among the harmful impacts [26]. More extreme weather events cause physical and psychological harm. World health experts have concluded with “very high confidence” that climate change already contributes to the global burden of disease and premature death [26].

IPCC [26] projects the following trends, if global warming continue to increase, where only trends assigned very high confidence or high confidence are included: (i) increased malnutrition and consequent disorders, including those related to child growth and development, (ii) increased death, disease and injuries from heat waves, floods, storms, fires and droughts, (iii) increased cardio-respiratory morbidity and mortality associated with ground-level ozone. While IPCC also projects fewer deaths from cold, this positive effect is far outweighed by the negative ones.

Growing awareness of the consequences of human-caused climate change triggers anxiety and feelings of helplessness [136]–[137]. Children, already susceptible to age-related insecurities, face additional destabilizing insecurities from questions about how they will cope with future climate change [138]–[139]. Exposure to media ensures that children cannot escape hearing that their future and that of other species is at stake, and that the window of opportunity to avoid dramatic climate impacts is closing. The psychological health of our children is a priority, but denial of the truth exposes our children to even greater risk.

Health impacts of climate change are in addition to direct effects of air and water pollution. A clear illustration of direct effects of fossil fuels on human health was provided by an inadvertent experiment in China during the 1950–1980 period of central planning, when free coal for winter heating was provided to North China but not to the rest of the country. Analysis of the impact was made [140] using the most comprehensive data file ever compiled on mortality and air pollution in any developing country. A principal conclusion was that the 500 million residents of North China experienced during the 1990s a loss of more than 2.5 billion life years owing to the added air pollution, and an average reduction in life expectancy of 5.5 years. The degree of air pollution in China exceeded that in most of the world, yet assessments of total health effects must also include other fossil fuel caused air and water pollutants, as discussed in the following section on ecology and the environment.

The Text S1 has further discussion of health impacts of climate change.

### Ecology and the Environment

The ecological impact of fossil fuel mining increases as the largest, easiest to access, resources are depleted [141]. A constant fossil fuel production rate requires increasing energy input, but also use of more land, water, and diluents, with the production of more waste [142]. The increasing ecological and environmental impact of a given amount of useful fossil fuel energy is a relevant consideration in assessing alternative energy strategies.

Coal mining has progressively changed from predominantly underground mining to surface mining [143], including mountaintop removal with valley fill, which is now widespread in the Appalachian ecoregion in the United States. Forest cover and topsoil are removed, explosives are used to break up rocks to access coal, and the excess rock is pushed into adjacent valleys, where it buries existing streams. Burial of headwater streams causes loss of ecosystems that are important for nutrient cycling and production of organic matter for downstream food webs [144]. The surface alterations lead to greater storm runoff [145] with likely impact on downstream flooding. Water emerging from valley fills contain toxic solutes that have been linked to declines in watershed biodiversity [146]. Even with mine-site reclamation intended to restore pre-mined surface conditions, mine-derived chemical constituents are found in domestic well water [147]. Reclaimed areas, compared with unmined areas, are found to have increased soil density with decreased organic and nutrient content, and with reduced water infiltration rates [148]. Reclaimed areas have been found to produce little if any regrowth of woody vegetation even after 15 years [149], and, although this deficiency might be addressed via more effective reclamation methods, there remains a likely significant loss of carbon storage [149].

Oil mining has an increasing ecological footprint per unit delivered energy because of the decreasing size of new fields and their increased geographical dispersion; transit distances are greater and wells are deeper, thus requiring more energy input [145]. Useful quantitative measures of the increasing ecological impacts are provided by the history of oil development in Alberta, Canada for production of both conventional oil and tar sands development. The area of land required per barrel of produced oil increased by a factor of 12 between 1955 and 2006 [150] leading to ecosystem fragmentation by roads and pipelines needed to support the wells [151]. Additional escalation of the mining impact occurs as conventional oil mining is supplanted by tar sands development, with mining and land disturbance from the latter producing land use-related greenhouse gas emissions as much as 23 times greater than conventional oil production per unit area [152], but with substantial variability and uncertainty [152]–[153]. Much of the tar sands bitumen is extracted through surface mining that removes the “overburden” (i.e., boreal forest ecosystems) and tar sand from large areas to a depth up to 100 m, with ecological impacts downstream and in the mined area [154]. Although mined areas are supposed to be reclaimed, as in the case of mountaintop removal, there is no expectation that the ecological value of reclaimed areas will be equivalent to predevelopment condition [141], [155]. Landscape changes due to tar sands mining and reclamation cause a large loss of peatland and stored carbon, while also significantly reducing carbon sequestration potential [156]. Lake sediment cores document increased chemical pollution of ecosystems during the past several decades traceable to tar sands development [157] and snow and water samples indicate that recent levels of numerous pollutants exceeded local and national criteria for protection of aquatic organisms [158].

Gas mining by unconventional means has rapidly expanded in recent years, without commensurate understanding of the ecological, environmental and human health consequences [159]. The predominant approach is hydraulic fracturing (“fracking”) of deep shale formations via injection of millions of gallons of water, sand and toxic chemicals under pressure, thus liberating methane [155], [160]. A large fraction of the injected water returns to the surface as wastewater containing high concentrations of heavy metals, oils, greases and soluble organic compounds [161]. Management of this wastewater is a major technical challenge, especially because the polluted waters can continue to backflow from the wells for many years [161]. Numerous instances of groundwater and river contamination have been cited [162]. High levels of methane leakage from fracking have been found [163], as well as nitrogen oxides and volatile organic compounds [159]. Methane leaks increase the climate impact of shale gas, but whether the leaks are sufficient to significantly alter the climate forcing by total natural gas development is uncertain [164]. Overall, environmental and ecologic threats posed by unconventional gas extraction are uncertain because of limited research, however evidence for groundwater pollution on both local and river basin scales is a major concern [165].

Today, with cumulative carbon emissions ∼370 GtC from all fossil fuels, we are at a point of severely escalating ecological and environmental impacts from fossil fuel use and fossil fuel mining, as is apparent from the mountaintop removal for coal, tar sands extraction of oil, and fracking for gas. The ecological and environmental implications of scenarios with carbon emissions of 1000 GtC or greater, as discussed below, would be profound and should influence considerations of appropriate energy strategies.

### Summary: Climate Impacts

Climate impacts accompanying global warming of 2°C or more would be highly deleterious. Already there are numerous indications of substantial effects in response to warming of the past few decades. That warming has brought global temperature close to if not slightly above the prior range of the Holocene. We conclude that an appropriate target would be to keep global temperature at a level within or close to the Holocene range. Global warming of 2°C would be well outside the Holocene range and far into the dangerous range.

## Transient Climate Change

We must quantitatively relate fossil fuel emissions to global temperature in order to assess how rapidly fossil fuel emissions must be phased down to stay under a given temperature limit. Thus we must deal with both a transient carbon cycle and transient global climate change.

Global climate fluctuates stochastically and also responds to natural and human-made climate forcings [1], [166]. Forcings, measured in W/m^2^ averaged over the globe, are imposed perturbations of Earth’s energy balance caused by changing forcing agents such as solar irradiance and human-made greenhouse gases (GHGs). CO_2_ accounts for more than 80% of the added GHG forcing in the past 15 years [64], [167] and, if fossil fuel emissions continue at a high level, CO_2_ will be the dominant driver of future global temperature change.

We first define our method of calculating atmospheric CO_2_ as a function of fossil fuel emissions. We then define our assumptions about the potential for drawing down atmospheric CO_2_ via reforestation and increase of soil carbon, and we define fossil fuel emission reduction scenarios that we employ in our study. Finally we describe all forcings employed in our calculations of global temperature and the method used to simulate global temperature.

### Carbon Cycle and Atmospheric CO_2_

The carbon cycle defines the fate of CO_2_ injected into the air by fossil fuel burning [1], [168] as the additional CO_2_ distributes itself over time among surface carbon reservoirs: the atmosphere, ocean, soil, and biosphere. We use the dynamic-sink pulse-response function version of the well-tested Bern carbon cycle model [169], as described elsewhere [54], [170].

Specifically, we solve equations 3–6, 16–17, A.2.2, and A.3 of Joos et al. [169] using the same parameters and assumptions therein, except that initial (1850) atmospheric CO_2_ is assumed to be 285.2 ppm [167]. Historical fossil fuel CO_2_ emissions are from Boden et al. [5]. This Bern model incorporates non-linear ocean chemistry feedbacks and CO_2_ fertilization of the terrestrial biosphere, but it omits climate-carbon feedbacks, e.g., assuming static global climate and ocean circulation. Therefore our results should be regarded as conservative, especially for scenarios with large emissions.

A pulse of CO_2_ injected into the air decays by half in about 25 years as CO_2_ is taken up by the ocean, biosphere and soil, but nearly one-fifth is still in the atmosphere after 500 years (Fig. 4A). Eventually, over hundreds of millennia, weathering of rocks will deposit all of this initial CO_2_ pulse on the ocean floor as carbonate sediments [168].

Under equilibrium conditions a negative CO_2_ pulse, i.e., artificial extraction and storage of some CO_2_ amount, decays at about the same rate as a positive pulse (Fig. 4A). Thus if it is decided in the future that CO_2_ must be extracted from the air and removed from the carbon cycle (e.g., by storing it underground or in carbonate bricks), the impact on atmospheric CO_2_ amount will diminish in time. This occurs because carbon is exchanged among the surface carbon reservoirs as they move toward an equilibrium distribution, and thus, e.g., CO_2_ out-gassing by the ocean can offset some of the artificial drawdown. The CO_2_ extraction required to reach a given target atmospheric CO_2_ level therefore depends on the prior emission history and target timeframe, but the amount that must be extracted substantially exceeds the net reduction of the atmospheric CO_2_ level that will be achieved. We clarify this matter below by means of specific scenarios for capture of CO_2_.

It is instructive to see how fast atmospheric CO_2_ declines if fossil fuel emissions are instantly terminated (Fig. 4B). Halting emissions in 2015 causes CO_2_ to decline to 350 ppm at century’s end (Fig. 4B). A 20 year delay in halting emissions has CO_2_ returning to 350 ppm at about 2300. With a 40 year delay, CO_2_ does not return to 350 ppm until after 3000. These results show how difficult it is to get back to 350 ppm if emissions continue to grow for even a few decades.

*These results emphasize the urgency of initiating emissions reduction* [171]. As discussed above, keeping global climate close to the Holocene range requires a long-term atmospheric CO_2_ level of about 350 ppm or less, with other climate forcings similar to today’s levels. If emissions reduction had begun in 2005, reduction at 3.5%/year would have achieved 350 ppm at 2100. Now the requirement is at least 6%/year. Delay of emissions reductions until 2020 requires a reduction rate of 15%/year to achieve 350 ppm in 2100. If we assume only 50 GtC reforestation, and begin emissions reduction in 2013, the required reduction rate becomes about 9%/year.

### Reforestation and Soil Carbon

Of course fossil fuel emissions will not suddenly terminate. Nevertheless, it is not impossible to return CO_2_ to 350 ppm this century. Reforestation and increase of soil carbon can help draw down atmospheric CO_2_. Fossil fuels account for ∼80% of the CO_2_ increase from preindustrial time, with land use/deforestation accounting for 20% [1], [170], [172]–[173]. Net deforestation to date is estimated to be 100 GtC (gigatons of carbon) with ±50% uncertainty [172].

Complete restoration of deforested areas is unrealistic, yet 100 GtC carbon drawdown is conceivable because: (1) the human-enhanced atmospheric CO_2_ level increases carbon uptake by some vegetation and soils, (2) improved agricultural practices can convert agriculture from a CO_2_ ource into a CO_2_ sink [174], (3) biomass-burning power plants with CO_2_ capture and storage can contribute to CO_2_ drawdown.

Forest and soil storage of 100 GtC is challenging, but has other benefits. Reforestation has been successful in diverse places [175]. Minimum tillage with biological nutrient recycling, as opposed to plowing and chemical fertilizers, could sequester 0.4–1.2 GtC/year [176] while conserving water in soils, building agricultural resilience to climate change, and increasing productivity especially in smallholder rain-fed agriculture, thereby reducing expansion of agriculture into forested ecosystems [177]–[178]. Net tropical deforestation may have decreased in the past decade [179], but because of extensive deforestation in earlier decades [170], [172]–[173], [180]–[181] there is a large amount of land suitable for reforestation [182].

Use of bioenergy to draw down CO_2_ should employ feedstocks from residues, wastes, and dedicated energy crops that do not compete with food crops, thus avoiding loss of natural ecosystems and cropland [183]–[185]. Reforestation competes with agricultural land use; land needs could decline by reducing use of animal products, as livestock now consume more than half of all crops [186].

Our reforestation scenarios assume that today’s net deforestation rate (∼1 GtC/year; see [54]) will stay constant until 2020, then linearly decrease to zero by 2030, followed by sinusoidal 100 GtC biospheric carbon storage over 2031–2080. Alternative timings do not alter conclusions about the potential to achieve a given CO_2_ level such as 350 ppm.

### Emission Reduction Scenarios

A 6%/year decrease of fossil fuel emissions beginning in 2013, with 100 GtC reforestation, achieves a CO_2_ decline to 350 ppm near the end of this century (Fig. 5A). Cumulative fossil fuel emissions in this scenario are ∼129 GtC from 2013 to 2050, with an additional 14 GtC by 2100. If our assumed land use changes occur a decade earlier, CO_2_ returns to 350 ppm several years earlier; however that has negligible effect on the maximum global temperature calculated below.

Delaying fossil fuel emission cuts until 2020 (with 2%/year emissions growth in 2012–2020) causes CO_2_ to remain above 350 ppm (with associated impacts on climate) until 2300 (Fig. 5B). If reductions are delayed until 2030 or 2050, CO_2_ remains above 350 ppm or 400 ppm, respectively, until well after 2500.

We conclude that it is urgent that large, long-term emission reductions begin soon. Even if a 6%/year reduction rate and 500 GtC are not achieved, it makes a huge difference when reductions begin. There is no practical justification for why emissions necessarily must even approach 1000 GtC.

### Climate Forcings

**Figure 6:**
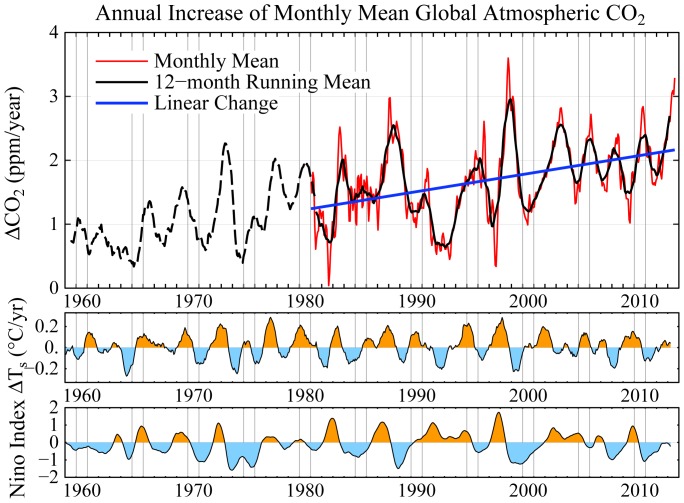
Annual increase of CO_2_ based on data from the NOAA Earth System Research Laboratory [188]. Prior to 1981 the CO_2_ change is based on only Mauna Loa, Hawaii. Temperature changes in lower diagram are 12-month running means for the globe and Niño3.4 area [16].

Atmospheric CO_2_ and other GHGs have been well-measured for the past half century, allowing accurate calculation of their climate forcing. The growth rate of the GHG forcing has declined moderately since its peak values in the 1980s, as the growth rate of CH_4_ and chlorofluorocarbons has slowed [187]. Annual changes of CO_2_ are highly correlated with the El Niño cycle (Fig. 6). Two strong La Niñas in the past five years have depressed CO_2_ growth as well as the global warming rate (Fig. 3). The CO_2_ growth rate and warming rate can be expected to increase as we move into the next El Niño, with the CO_2_ growth already reaching 3 ppm/year in mid-2013 [188]. The CO_2_ climate forcing does not increase as rapidly as the CO_2_ amount because of partial saturation of CO_2_ absorption bands [75]. The GHG forcing is now increasing at a rate of almost 0.4 W/m^2^ per decade [187].

**Figure 7:**
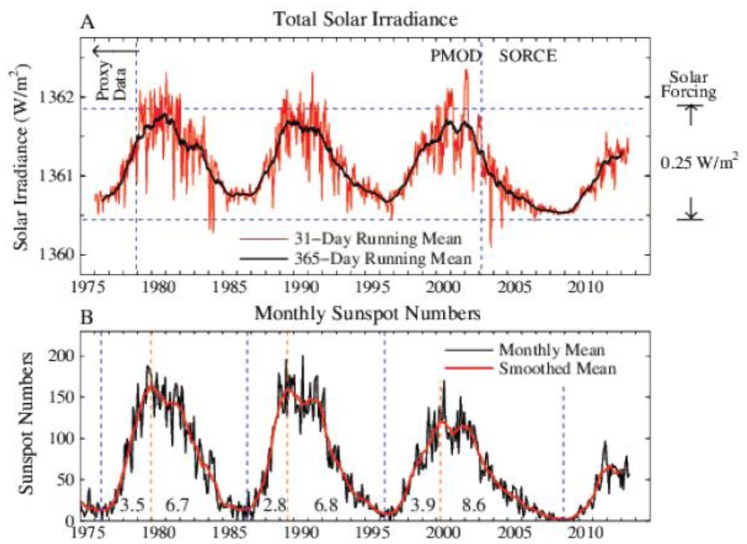
Solar irradiance and sunspot number in the era of satellite data (see text). Left scale is the energy passing through an area perpendicular to Sun-Earth line. Averaged over Earth’s surface the absorbed solar energy is ∼240 W/m^2^, so the full amplitude of measured solar variability is ∼0.25 W/m^2^.

Solar irradiance variations are sometimes assumed to be the most likely natural driver of climate change. Solar irradiance has been measured from satellites since the late 1970s (Fig. 7). These data are from a composite of several satellite-measured time series. Data through 28 February 2003 are from [189] and Physikalisch Meteorologisches Observatorium Davos, World Radiation Center. Subsequent update is from University of Colorado Solar Radiation & Climate Experiment (SORCE). Data sets are concatenated by matching the means over the first 12 months of SORCE data. Monthly sunspot numbers (Fig. 7) support the conclusion that the solar irradiance in the current solar cycle is significantly lower than in the three preceding solar cycles. Amplification of the direct solar forcing is conceivable, e.g., through effects on ozone or atmospheric condensation nuclei, but empirical data place a factor of two upper limit on the amplification, with the most likely forcing in the range 100–120% of the directly measured solar irradiance change [64].

Recent reduced solar irradiance (Fig. 7) may have decreased the forcing over the past decade by about half of the full amplitude of measured irradiance variability, thus yielding a negative forcing of, say, − 0.12 W/m^2^. This compares with a decadal increase of the GHG forcing that is positive and about three times larger in magnitude. Thus the solar forcing is not negligible and might partially account for the slowdown in global warming in the past decade [17]. However, we must (1) compare the solar forcing with the net of other forcings, which enhances the importance of solar change, because the net forcing is smaller than the GHG forcing, and (2) consider forcing changes on longer time scales, which greatly diminishes the importance of solar change, because solar variability is mainly oscillatory.

**Figure 8:**
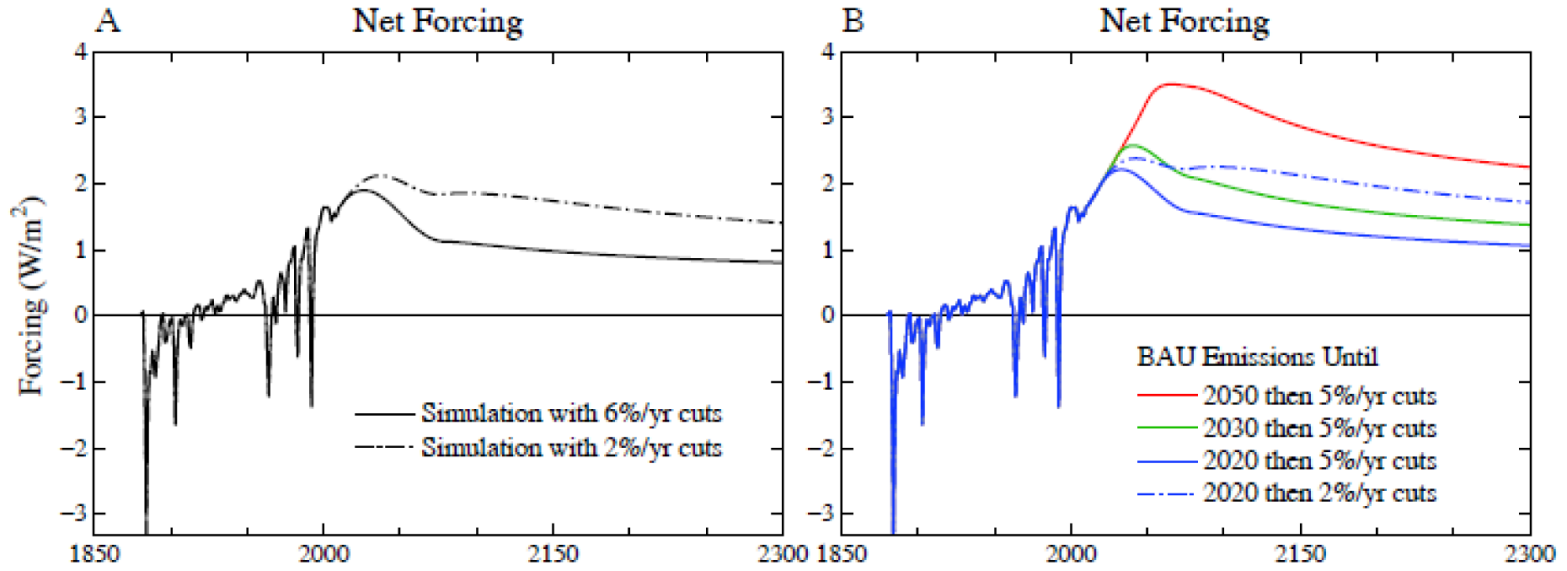
Climate forcings employed in our six main scenarios. Forcings through 2010 are as in [64].

Human-made tropospheric aerosols, which arise largely from fossil fuel use, cause a substantial negative forcing. As noted above, two independent analyses [64], [72] yield a total (direct plus indirect) aerosol forcing in the past decade of about −1.5 W/m^2^, half the magnitude of the GHG forcing and opposite in sign. That empirical aerosol forcing assessment for the past decade is consistent with the climate forcings scenario (Fig. 8) that we use for the past century in the present and prior studies [64], [190]. Supplementary Table S1 specifies the historical forcings and Table S2 gives several scenarios for future forcings.

### Future Climate Forcings

Future global temperature change should depend mainly on atmospheric CO_2_, at least if fossil fuel emissions remain high. Thus to provide the clearest picture of the CO_2_ effect, we approximate the net future change of human-made non-CO_2_ forcings as zero and we exclude future changes of natural climate forcings, such as solar irradiance and volcanic aerosols. Here we discuss possible effects of these approximations.

Uncertainties in non-CO_2_ forcings concern principally solar, aerosol and other GHG forcings. Judging from the sunspot numbers (Fig. 7B and [191]) for the past four centuries, the current solar cycle is almost as weak as the Dalton Minimum of the late 18th century. Conceivably irradiance could decline further to the level of the Maunder Minimum of the late 17th century [192]–[193]. For our simulation we choose an intermediate path between recovery to the level before the current solar cycle and decline to a still lower level. Specifically, we keep solar irradiance fixed at the reduced level of 2010, which is probably not too far off in either direction. Irradiance in 2010 is about 0.1 W/m^2^ less than the mean of the prior three solar cycles, a decrease of forcing that would be restored by the CO_2_ increase within 3–4 years at its current growth rate. Extensive simulations [17], [194] confirm that the effect of solar variability is small compared with GHGs if CO_2_ emissions continue at a high level. However, solar forcing can affect the magnitude and detection of near-term warming. Also, if rapidly declining GHG emissions are achieved, changes of solar forcing will become relatively more important.

Aerosols present a larger uncertainty. Expectations of decreases in large source regions such as China [195] may be counteracted by aerosol increases other places as global population continues to increase. Our assumption of unchanging human-made aerosols could be substantially off in either direction. For the sake of interpreting on-going and future climate change it is highly desirable to obtain precise monitoring of the global aerosol forcing [73].

Non-CO_2_ GHG forcing has continued to increase at a slow rate since 1995 (Fig. 6 in [64]). A desire to constrain climate change may help reduce emissions of these gases in the future. However, it will be difficult to prevent or fully offset positive forcing from increasing N_2_O, as its largest source is associated with food production and the world’s population is continuing to rise.

On the other hand, we are also probably underestimating a negative aerosol forcing, e.g., because we have not included future volcanic aerosols. Given the absence of large volcanic eruptions in the past two decades (the last one being Mount Pinatubo in 1991), multiple volcanic eruptions would cause a cooling tendency [196] and reduce heat storage in the ocean [197].

Overall, we expect the errors due to our simple approximation of non-CO_2_ forcings to be partially off-setting. Specifically, we have likely underestimated a positive forcing by non-CO_2_ GHGs, while also likely underestimating a negative aerosol forcing.

Note that uncertainty in forcings is partly obviated via the focus on Earth’s energy imbalance in our analysis. The planet’s energy imbalance is an integrative quantity that is especially useful for a case in which some of the forcings are uncertain or unmeasured. Earth’s measured energy imbalance includes the effects of all forcings, whether they are measured or not.

### Simulations of Future Global Temperature

We calculate global temperature change for a given CO_2_ scenario using a climate response function (Table S3) that accurately replicates results from a global climate model with sensitivity 3°C for doubled CO_2_ [64]. A best estimate of climate sensitivity close to 3°C for doubled CO_2_ has been inferred from paleoclimate data [51]–[52]. This empirical climate sensitivity is generally consistent with that of global climate models [1], but the empirical approach makes the inferred high sensitivity more certain and the quantitative evaluation more precise. Because this climate sensitivity is derived from empirical data on how Earth responded to past changes of boundary conditions, including atmospheric composition, our conclusions about limits on fossil fuel emissions can be regarded as largely independent of climate models.

The detailed temporal and geographical response of the climate system to the rapid human-made change of climate forcings is not well-constrained by empirical data, because there is no faithful paleoclimate analog. Thus climate models necessarily play an important role in assessing practical implications of climate change. Nevertheless, it is possible to draw important conclusions with transparent computations. A simple response function (Green’s function) calculation [64] yields an estimate of global mean temperature change in response to a specified time series for global climate forcing. This approach accounts for the delayed response of the climate system caused by the large thermal inertia of the ocean, yielding a global mean temporal response in close accord with that obtained from global climate models.

Tables S1 and S2 in Supporting Information give the forcings we employ and Table S3 gives the climate response function for our Green’s function calculation, defined by equation 2 of [64]. The Green’s function is driven by the net forcing, which, with the response function, is sufficient information for our results to be reproduced. However, we also include the individual forcings in Table S1, in case researchers wish to replace specific forcings or use them for other purposes.

**Figure 9:**
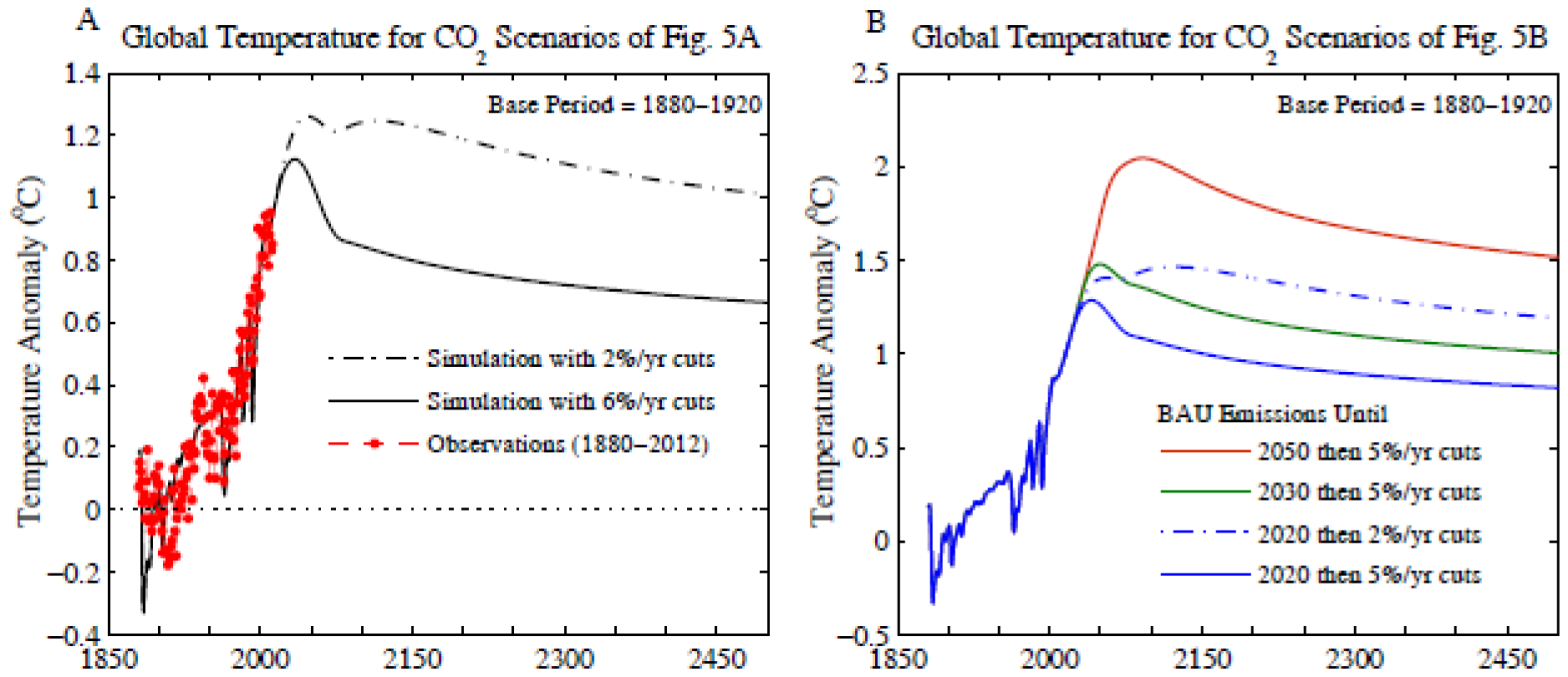
Simulated global temperature relative to 1880–1920 mean for CO_2_ scenarios of Figure 5.

Simulated global temperature (Fig. 9) is for CO_2_ scenarios of Fig. 5. Peak global warming is ∼1.1°C, declining to less than 1°C by mid-century, if CO_2_ emissions are reduced 6%/year beginning in 2013. In contrast, warming reaches 1.5°C and stays above 1°C until after 2400 if emissions continue to increase until 2030, even though fossil fuel emissions are phased out rapidly (5%/year) after 2030 and 100 GtC reforestation occurs during 2030–2080. If emissions continue to increase until 2050, simulated warming exceeds 2°C well into the 22^nd^ century.

Increased global temperature persists for many centuries after the climate forcing declines, because of the thermal inertia of the ocean [198]. Some temperature reduction is possible if the climate forcing is reduced rapidly, before heat has penetrated into the deeper ocean. Cooling by a few tenths of a degree in Fig. 9 is a result mainly of the 100 GtC biospheric uptake of CO_2_ during 2030–2080. Note the longevity of the warming, especially if emissions reduction is as slow as 2%/year, which might be considered to be a rapid rate of reduction.

The temporal response of the real world to the human-made climate forcing could be more complex than suggested by a simple response function calculation, especially if rapid emissions growth continues, yielding an unprecedented climate forcing scenario. For example, if ice sheet mass loss becomes rapid, it is conceivable that the cold fresh water added to the ocean could cause regional surface cooling [199], perhaps even at a point when sea level rise has only reached a level of the order of a meter [200]. However, any uncertainty in the surface thermal response this century due to such phenomena has little effect on our estimate of the dangerous level of emissions. The long lifetime of the fossil fuel carbon in the climate system and the persistence of ocean warming for millennia [201] provide sufficient time for the climate system to achieve full response to the fast feedback processes included in the 3°C climate sensitivity.

Indeed, the long lifetime of fossil fuel carbon in the climate system and persistence of the ocean warming ensure that “slow” feedbacks, such as ice sheet disintegration, changes of the global vegetation distribution, melting of permafrost, and possible release of methane from methane hydrates on continental shelves, would also have time to come into play. Given the unprecedented rapidity of the human-made climate forcing, it is difficult to establish how soon slow feedbacks will become important, but clearly slow feedbacks should be considered in assessing the “dangerous” level of global warming, as discussed in the next section.

## Danger of Initiating Uncontrollable Climate Change

Our calculated global warming as a function of CO_2_ amount is based on equilibrium climate sensitivity 3°C for doubled CO_2_. That is the central climate sensitivity estimate from climate models [1], and it is consistent with climate sensitivity inferred from Earth’s climate history [51]–[52]. However, this climate sensitivity includes only the effects of fast feedbacks of the climate system, such as water vapor, clouds, aerosols, and sea ice. Slow feedbacks, such as change of ice sheet area and climate-driven changes of greenhouse gases, are not included.

### Slow Climate Feedbacks and Irreversible Climate Change

Excluding slow feedbacks was appropriate for simulations of the past century, because we know the ice sheets were stable then and our climate simulations used observed greenhouse gas amounts that included any contribution from slow feedbacks. However, we must include slow feedbacks in projections of warming for the 21^st^ century and beyond. Slow feedbacks are important because they affect climate sensitivity and because their instigation is related to the danger of passing “points of no return”, beyond which irreversible consequences become inevitable, out of humanity’s control.

Antarctic and Greenland ice sheets present the danger of change with consequences that are irreversible on time scales important to society [1]. These ice sheets required millennia to grow to their present sizes. If ice sheet disintegration reaches a point such that the dynamics and momentum of the process take over, at that point reducing greenhouse gases may be unable to prevent major ice sheet mass loss, sea level rise of many meters, and worldwide loss of coastal cities – a consequence that is irreversible for practical purposes. Interactions between the ocean and ice sheets are particularly important in determining ice sheet changes, as a warming ocean can melt the ice shelves, the tongues of ice that extend from the ice sheets into the ocean and buttress the large land-based ice sheets [92], [202]–[203]. Paleoclimate data for sea level change indicate that sea level changed at rates of the order of a meter per century [81]–[83], even at times when the forcings driving climate change were far weaker than the human-made forcing. Thus, because ocean warming is persistent for centuries, there is a danger that large irreversible change could be initiated by excessive ocean warming.

**Figure 10:**
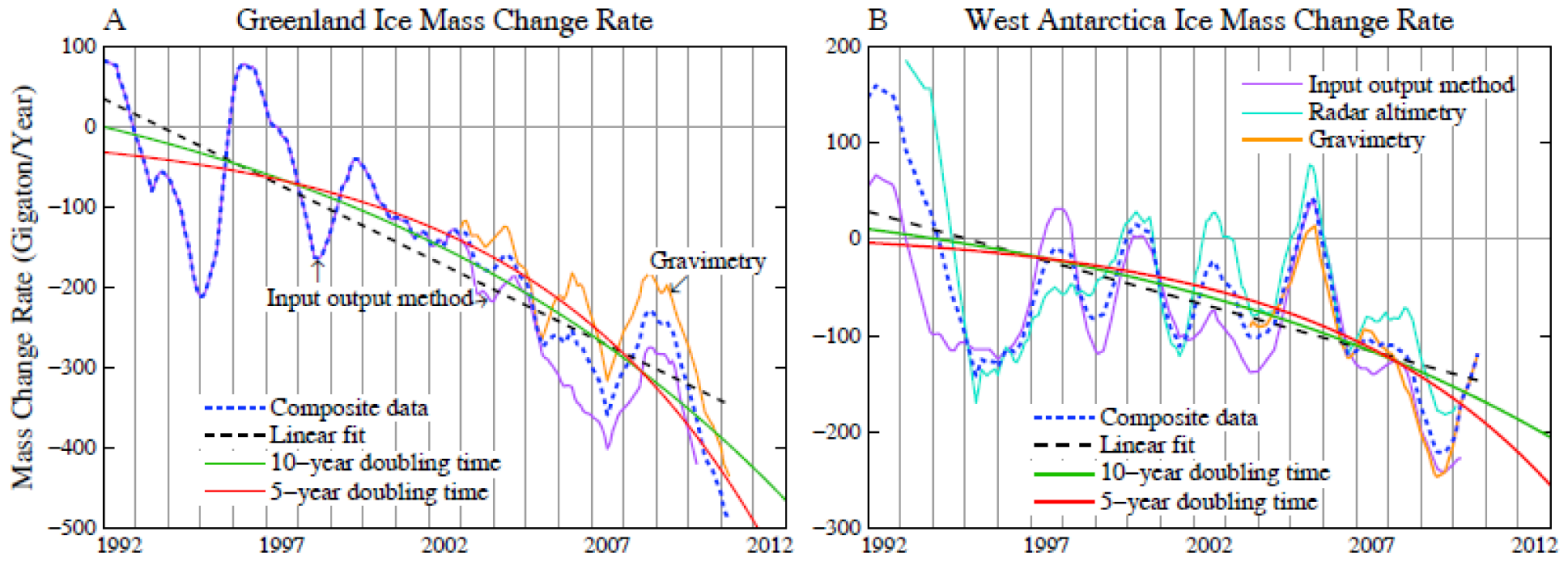
Annual Greenland and West Antarctic ice mass changes as estimated via alternative methods. Data were read from Figure 4 of Shepherd et al. [23] and averaged over the available records.

Paleoclimate data are not as helpful for defining the likely rate of sea level rise in coming decades, because there is no known case of growth of a positive (warming) climate forcing as rapid as the anthropogenic change. The potential for unstable ice sheet disintegration is controversial, with opinion varying from likely stability of even the (marine) West Antarctic ice sheet [94] to likely rapid non-linear response extending up to multi-meter sea level rise [97]–[98]. Data for the modern rate of annual ice sheet mass changes indicate an accelerating rate of mass loss consistent with a mass loss doubling time of a decade or less (Fig. 10). However, we do not know the functional form of ice sheet response to a large persistent climate forcing. Longer records are needed for empirical assessment of this ostensibly nonlinear behavior.

Greenhouse gas amounts in the atmosphere, most importantly CO_2_ and CH_4_, change in response to climate change, i.e., as a feedback, in addition to the immediate gas changes from human-caused emissions. As the ocean warms, for example, it releases CO_2_ to the atmosphere, with one principal mechanism being the simple fact that the solubility of CO_2_ decreases as the water temperature rises [204]. We also include in the category of slow feedbacks the global warming spikes, or “hyperthermals”, that have occurred a number of times in Earth’s history during the course of slower global warming trends. The mechanisms behind these hyperthermals are poorly understood, as discussed below, but they are characterized by the injection into the surface climate system of a large amount of carbon in the form of CH_4_ and/or CO_2_ on the time scale of a millennium [205]–[207]. The average rate of injection of carbon into the climate system during these hyperthermals was slower than the present human-made injection of fossil fuel carbon, yet it was faster than the time scale for removal of carbon from the surface reservoirs via the weathering process [3], [208], which is tens to hundreds of thousands of years.

Methane hydrates – methane molecules trapped in frozen water molecule cages in tundra and on continental shelves – and organic matter such as peat locked in frozen soils (permafrost) are likely mechanisms in the past hyperthermals, and they provide another climate feedback with the potential to amplify global warming if large scale thawing occurs [209]–[210]. Paleoclimate data reveal instances of rapid global warming, as much as 5–6°C, as a sudden additional warming spike during a longer period of gradual warming [see Text S1]. The candidates for the carbon injected into the climate system during those warmings are methane hydrates on continental shelves destabilized by sea floor warming [211] and carbon released from frozen soils [212]. As for the present, there are reports of methane release from thawing permafrost on land [213] and from sea-bed methane hydrate deposits [214], but amounts so far are small and the data are snapshots that do not prove that there is as yet a temporal increase of emissions.

There is a possibility of rapid methane hydrate or permafrost emissions in response to warming, but that risk is largely unquantified [215]. The time needed to destabilize large methane hydrate deposits in deep sediments is likely millennia [215]. Smaller but still large methane hydrate amounts below shallow waters as in the Arctic Ocean are more vulnerable; the methane may oxidize to CO_2_ in the water, but it will still add to the long-term burden of CO_2_ in the carbon cycle. Terrestrial permafrost emissions of CH_4_ and CO_2_ likely can occur on a time scale of a few decades to several centuries if global warming continues [215]. These time scales are within the lifetime of anthropogenic CO_2_, and thus these feedbacks must be considered in estimating the dangerous level of global warming. Because human-made warming is more rapid than natural long-term warmings in the past, there is concern that methane hydrate or peat feedbacks could be more rapid than the feedbacks that exist in the paleoclimate record.

Climate model studies and empirical analyses of paleoclimate data can provide estimates of the amplification of climate sensitivity caused by slow feedbacks, excluding the singular mechanisms that caused the hyperthermal events. Model studies for climate change between the Holocene and the Pliocene, when Earth was about 3°C warmer, find that slow feedbacks due to changes of ice sheets and vegetation cover amplified the fast feedback climate response by 30–50% [216]. These same slow feedbacks are estimated to amplify climate sensitivity by almost a factor of two for the climate change between the Holocene and the nearly ice-free climate state that existed 35 million years ago [54].

### Implication for Carbon Emissions Target

Evidence presented under Climate Impacts above makes clear that 2°C global warming would have consequences that can be described as disastrous. Multiple studies [12], [198], [201] show that the warming would be very long lasting. The paleoclimate record and changes underway in the Arctic and on the Greenland and Antarctic ice sheets with only today’s warming imply that sea level rise of several meters could be expected. Increased climate extremes, already apparent at 0.8°C warming [46], would be more severe. Coral reefs and associated species, already stressed with current conditions [40], would be decimated by increased acidification, temperature and sea level rise. More generally, humanity and nature, the modern world as we know it, is adapted to the Holocene climate that has existed more than 10,000 years. Warming of 1°C relative to 1880–1920 keeps global temperature close to the Holocene range, but warming of 2°C, to at least the Eemian level, could cause major dislocations for civilization.

However, distinctions between pathways aimed at ∼1°C and 2°C warming are much greater and more fundamental than the numbers 1°C and 2°C themselves might suggest. These fundamental distinctions make scenarios with 2°C or more global warming far more dangerous; so dangerous, we suggest, that aiming for the 2°C pathway would be foolhardy.

First, most climate simulations, including ours above and those of IPCC [1], do not include slow feedbacks such as reduction of ice sheet size with global warming or release of greenhouse gases from thawing tundra. These exclusions are reasonable for a ∼1°C scenario, because global temperature barely rises out of the Holocene range and then begins to subside. In contrast, global warming of 2°C or more is likely to bring slow feedbacks into play. Indeed, it is slow feedbacks that cause long-term climate sensitivity to be high in the empirical paleoclimate record [51]–[52]. The lifetime of fossil fuel CO_2_ in the climate system is so long that it must be assumed that these slow feedbacks will occur if temperature rises well above the Holocene range.

Second, scenarios with 2°C or more warming necessarily imply expansion of fossil fuels into sources that are harder to get at, requiring greater energy using extraction techniques that are increasingly invasive, destructive and polluting. Fossil fuel emissions through 2012 total ∼370 GtC (Fig. 2). If subsequent emissions decrease 6%/year, additional emissions are ∼130 GtC, for a total ∼500 GtC fossil fuel emissions. This 130 GtC can be obtained mainly from the easily extracted conventional oil and gas reserves (Fig. 2), with coal use rapidly phased out and unconventional fossil fuels left in the ground. In contrast, 2°C scenarios have total emissions of the order of 1000 GtC. The required additional fossil fuels will involve exploitation of tar sands, tar shale, hydrofracking for oil and gas, coal mining, drilling in the Arctic, Amazon, deep ocean, and other remote regions, and possibly exploitation of methane hydrates. Thus 2°C scenarios result in more CO_2_ per unit useable energy, release of substantial CH_4_ via the mining process and gas transportation, and release of CO_2_ and other gases via destruction of forest “overburden” to extract subterranean fossil fuels.

Third, with our ∼1°C scenario it is more likely that the biosphere and soil will be able to sequester a substantial portion of the anthropogenic fossil fuel CO_2_ carbon than in the case of 2°C or more global warming. Empirical data for the CO_2_ “airborne fraction”, the ratio of observed atmospheric CO_2_ increase divided by fossil fuel CO_2_ emissions, show that almost half of the emissions is being taken up by surface (terrestrial and ocean) carbon reservoirs [187], despite a substantial but poorly measured contribution of anthropogenic land use (deforestation and agriculture) to airborne CO_2_ [179], [216]. Indeed, uptake of CO_2_ by surface reservoirs has at least kept pace with the rapid growth of emissions [187]. Increased uptake in the past decade may be a consequence of a reduced rate of deforestation [217] and fertilization of the biosphere by atmospheric CO_2_ and nitrogen deposition [187]. With the stable climate of the ∼1°C scenario it is plausible that major efforts in reforestation and improved agricultural practices [15], [173], [175]–[177], with appropriate support provided to developing countries, could take up an amount of carbon comparable to the 100 GtC in our ∼1°C scenario. On the other hand, with warming of 2°C or more, carbon cycle feedbacks are expected to lead to substantial additional atmospheric CO_2_ [218]–[219], perhaps even making the Amazon rainforest a source of CO_2_ [219]–[220].

Fourth, a scenario that slows and then reverses global warming makes it possible to reduce other greenhouse gases by reducing their sources [75], [221]. The most important of these gases is CH_4_, whose reduction in turn reduces tropospheric O_3_ and stratospheric H_2_O. In contrast, chemistry modeling and paleoclimate records [222] show that trace gases increase with global warming, making it unlikely that overall atmospheric CH_4_ will decrease even if a decrease is achieved in anthropogenic CH_4_ sources. Reduction of the amount of atmospheric CH_4_ and related gases is needed to counterbalance expected forcing from increasing N_2_O and decreasing sulfate aerosols.

Now let us compare the 1°C (500 GtC fossil fuel emissions) and the 2°C (1000 GtC fossil fuel emissions) scenarios. Global temperature in 2100 would be close to 1°C in the 500 GtC scenario, and it is less than 1°C if 100 GtC uptake of carbon by the biosphere and soil is achieved via improved agricultural and forestry practices (Fig. 9). In contrast, the 1000 GtC scenario, although nominally designed to yield a fast-feedback climate response of ∼ 2°C, would yield a larger eventual warming because of slow feedbacks, probably at least 3°C.

### Danger of Uncontrollable Consequences

Inertia of the climate system reduces the near-term impact of human-made climate forcings, but that inertia is not necessarily our friend. One implication of the inertia is that climate impacts “in the pipeline” may be much greater than the impacts that we presently observe. Slow climate feedbacks add further danger of climate change running out of humanity’s control. The response time of these slow feedbacks is uncertain, but there is evidence that some of these feedbacks already are underway, at least to a minor degree. Paleoclimate data show that on century and millennial time scales the slow feedbacks are predominately amplifying feedbacks.

The inertia of energy system infrastructure, i.e., the time required to replace fossil fuel energy systems, will make it exceedingly difficult to avoid a level of atmospheric CO_2_ that would eventually have highly undesirable consequences. The danger of uncontrollable and irreversible consequences necessarily raises the question of whether it is feasible to extract CO_2_ from the atmosphere on a large enough scale to affect climate change.

## Carbon Extraction

We have shown that extraordinarily rapid emission reductions are needed to stay close to the 1°C scenario. In absence of extraordinary actions, it is likely that growing climate disruptions will lead to a surge of interest in “geo-engineering” designed to minimize human-made climate change [223]. Such efforts must remove atmospheric CO_2_, if they are to address direct CO_2_ effects such as ocean acidification as well as climate change. Schemes such as adding sulfuric acid aerosols to the stratosphere to reflect sunlight [224], an attempt to mask one pollutant with another, is a temporary band-aid for a problem that will last for millennia; besides it fails to address ocean acidification and may have other unintended consequences [225].

### Potential for Carbon Extraction

At present there are no proven technologies capable of large-scale air capture of CO_2_. It has been suggested that, with strong research and development support and industrial scale pilot projects sustained over decades, costs as low as ∼$500/tC may be achievable [226]. Thermodynamic constraints [227] suggest that this cost estimate may be low. An assessment by the American Physical Society [228] argues that the lowest currently achievable cost, using existing approaches, is much greater ($600/tCO_2_ or $2200/tC).

The cost of capturing 50 ppm of CO_2_, at $500/tC (∼$135/tCO_2_), is ∼$50 trillion (1 ppm CO_2_ is ∼2.12 GtC), but more than $200 trillion for the price estimate of the American Physical Society study. Moreover, the resulting atmospheric CO_2_ reduction will ultimately be less than 50 ppm for the reasons discussed above. For example, let us consider the scenario of Fig. 5B in which emissions continue to increase until 2030 before decreasing at 5%/year – this scenario yields atmospheric CO_2_ of 410 ppm in 2100. Using our carbon cycle model we calculate that if we extract 100 ppm of CO_2_ from the air over the period 2030–2100 (10/7 ppm per year), say storing that CO_2_ in carbonate bricks, the atmospheric CO_2_ amount in 2100 will be reduced 52 ppm to 358 ppm, i.e., the reduction of airborne CO_2_ is about half of the amount extracted from the air and stored. The estimated cost of this 52 ppm CO_2_ reduction is $100–400 trillion.

The cost of CO_2_ capture and storage conceivably may decline in the future. Yet the practicality of carrying out such a program with alacrity in response to a climate emergency is dubious. Thus it may be appropriate to add a CO_2_ removal cost to the current price of fossil fuels, which would both reduce ongoing emissions and provide resources for future cleanup.

### Responsibility for Carbon Extraction

We focus on fossil fuel carbon, because of its long lifetime in the carbon cycle. Reversing the effects of deforestation is also important and there will need to be incentives to achieve increased carbon storage in the biosphere and soil, but the crucial requirement now is to limit the amount of fossil fuel carbon in the air.

**Figure 11:**
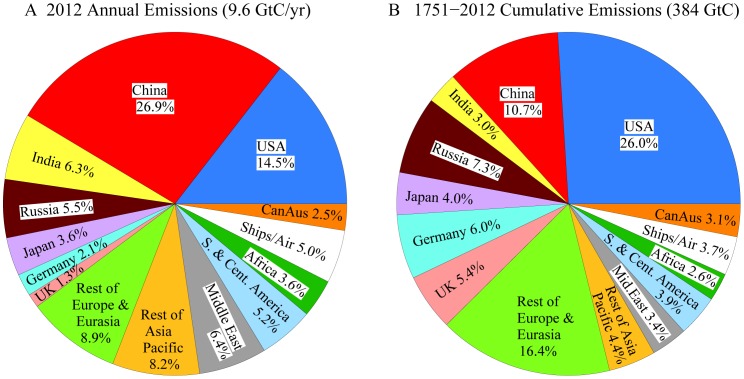
Fossil fuel CO_2_ emissions. (A) 2012 emissions by source region, and (B) cumulative 1751–2012 emissions. Results are an update of Fig. 10 of [190] using data from [5].

**Figure 12:**
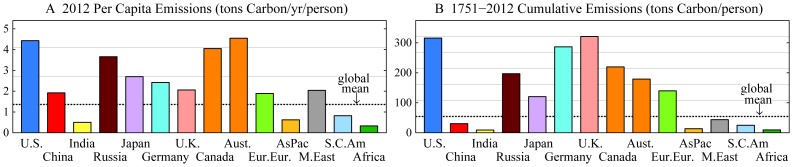
Per capita fossil fuel CO_2_ emissions. Countries, regions and data sources are the same as in Fig. 11. Horizontal lines are the global mean and multiples of the global mean.

The high cost of carbon extraction naturally raises the question of responsibility for excess fossil fuel CO_2_ in the air. China has the largest CO_2_ emissions today (Fig. 11A), but the global warming effect is closely proportional to cumulative emissions [190]. The United States is responsible for about one-quarter of cumulative emissions, with China next at about 10% (Fig. 11B). Cumulative responsibilities change rather slowly (compare Fig. 10 of 190). Estimated per capita emissions (Fig. 12) are based on population estimates for 2009–2011.

Various formulae might be devised to assign costs of CO_2_ air capture, should removal prove essential for maintaining acceptable climate. For the sake of estimating the potential cost, let us assume that it proves necessary to extract 100 ppm of CO_2_ (yielding a reduction of airborne CO_2_ of about 50 ppm) and let us assign each country the responsibility to clean up its fraction of cumulative emissions. Assuming a cost of $500/tC (∼$135/tCO_2_) yields a cost of $28 trillion for the United States, about $90,000 per individual. Costs would be slightly higher for a UK citizen, but less for other nations (Fig. 12B).

Cost of CO_2_ capture might decline, but the cost estimate used is more than a factor of four smaller than estimated by the American Physical Society [228] and 50 ppm is only a moderate reduction. The cost should also include safe permanent disposal of the captured CO_2_, which is a substantial mass. For the sake of scaling the task, note that one GtC, made into carbonate bricks, would produce the volume of ∼3000 Empire State buildings or ∼1200 Great Pyramids of Giza. Thus the 26 ppm assigned to the United States, if made into carbonate bricks, would be equivalent to the stone in 165,000 Empire State buildings or 66,000 Great Pyramids of Giza. This is not intended as a practical suggestion: carbonate bricks are not a good building material, and the transport and construction costs would be additional.

The point of this graphic detail is to make clear the magnitude of the cleanup task and potential costs, if fossil fuel emissions continue unabated. More useful and economic ways of removing CO_2_ may be devised with the incentive of a sufficient carbon price. For example, a stream of pure CO_2_ becomes available for capture and storage if biomass is used as the fuel for power plants or as feedstock for production of liquid hydrocarbon fuels. Such clean energy schemes and improved agricultural and forestry practices are likely to be more economic than direct air capture of CO_2_, but they must be carefully designed to minimize undesirable impacts and the amount of CO_2_ that can be extracted on the time scale of decades will be limited, thus emphasizing the need to limit the magnitude of the cleanup task.

## Policy Implications

Human-made climate change concerns physical sciences, but leads to implications for policy and politics. Conclusions from the physical sciences, such as the rapidity with which emissions must be reduced to avoid obviously unacceptable consequences and the long lag between emissions and consequences, lead to implications in social sciences, including economics, law and ethics. Intergovernmental climate assessments [1], [14] purposely are not policy prescriptive. Yet there is also merit in analysis and discussion of the full topic through the objective lens of science, i.e., “connecting the dots” all the way to policy implications.

### Energy and Carbon Pathways: A Fork in the Road

**Figure 13:**
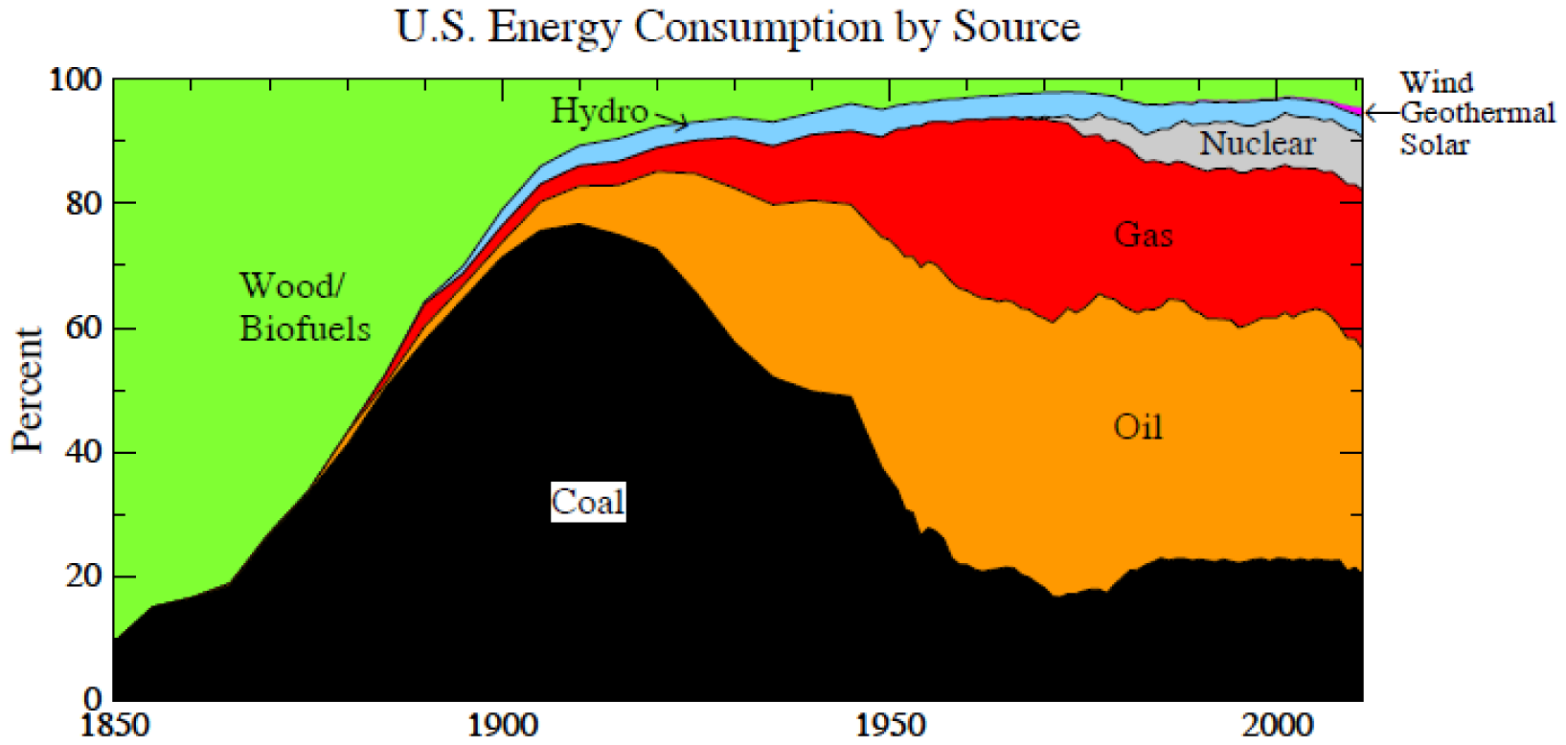
United States energy consumption [229].

**Figure 14:**
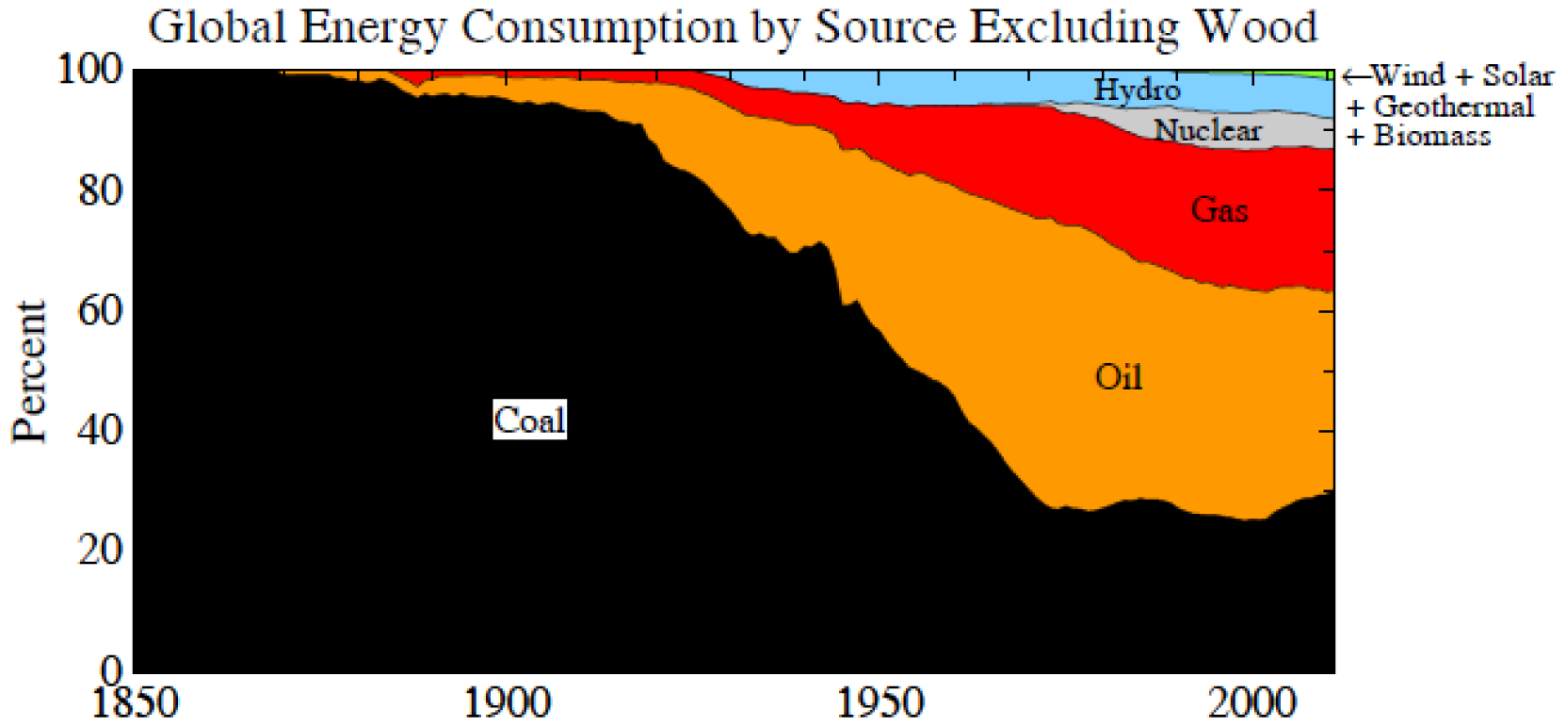
World energy consumption for indicated fuels, which excludes wood [4].

The industrial revolution began with wood being replaced by coal as the primary energy source. Coal provided more concentrated energy, and thus was more mobile and effective. We show data for the United States (Fig. 13) because of the availability of a long data record that includes wood [229]. More limited global records yield a similar picture [Fig. 14], the largest difference being global coal now at ∼30% compared with ∼20% in the United States. Economic progress and wealth generation were further spurred in the twentieth century by expansion into liquid and gaseous fossil fuels, oil and gas being transported and burned more readily than coal. Only in the latter part of the twentieth century did it become clear that long-lived combustion products from fossil fuels posed a global climate threat, as formally acknowledged in the 1992 Framework Convention on Climate Change [6]. However, efforts to slow emissions of the principal atmospheric gas driving climate change, CO_2_, have been ineffectual so far (Fig. 1).

Consequently, at present, as the most easily extracted oil and gas reserves are being depleted, we stand at a fork in the road to our energy and carbon future. Will we now feed our energy needs by pursuing difficult to extract fossil fuels, or will we pursue energy policies that phase out carbon emissions, moving on to the post fossil fuel era as rapidly as practical?

This is not the first fork encountered. Most nations agreed to the Framework Convention on Climate Change in 1992 [6]. Imagine if a bloc of countries favoring action had agreed on a common gradually rising carbon fee collected within each of country at domestic mines and ports of entry. Such nations might place equivalent border duties on products from nations not having a carbon fee and they could rebate fees to their domestic industry for export products to nations without an equivalent carbon fee. The legality of such a border tax adjustment under international trade law is untested, but is considered to be plausibly consistent with trade principles [230]. As the carbon fee gradually rose and as additional nations, for their own benefit, joined this bloc of nations, development of carbon-free energies and energy efficiency would have been spurred. If the carbon fee had begun in 1995, we calculate that global emissions would have needed to decline 2.1%/year to limit cumulative fossil fuel emissions to 500 GtC. A start date of 2005 would have required a reduction of 3.5%/year for the same result.

The task faced today is more difficult. Emissions reduction of 6%/year and 100 GtC storage in the biosphere and soils are needed to get CO_2_ back to 350 ppm, the approximate requirement for restoring the planet’s energy balance and stabilizing climate this century. Such a pathway is exceedingly difficult to achieve, given the current widespread absence of policies to drive rapid movement to carbon-free energies and the lifetime of energy infrastructure in place.

Yet we suggest that a pathway is still conceivable that could restore planetary energy balance on the century time scale. That path requires policies that spur technology development and provide economic incentives for consumers and businesses such that social tipping points are reached where consumers move rapidly to energy conservation and low carbon energies. Moderate overshoot of required atmospheric CO_2_ levels can possibly be counteracted via incentives for actions that more-or-less naturally sequester carbon. Developed countries, responsible for most of the excess CO_2_ in the air, might finance extensive efforts in developing countries to sequester carbon in the soil and in forest regrowth on marginal lands as described above. Burning sustainably designed biofuels in power plants, with the CO_2_ captured and sequestered, would also help draw down atmospheric CO_2_. This pathway would need to be taken soon, as the magnitude of such carbon extractions is likely limited and thus not a solution to unfettered fossil fuel use.

The alternative pathway, which the world seems to be on now, is continued extraction of all fossil fuels, including development of unconventional fossil fuels such as tar sands, tar shale, hydrofracking to extract oil and gas, and exploitation of methane hydrates. If that path (with 2%/year growth) continues for 20 years and is then followed by 3%/year emission reduction from 2033 to 2150, we find that fossil fuel emissions in 2150 would total 1022 GtC. Extraction of the excess CO_2_ from the air in this case would be very expensive and perhaps implausible, and warming of the ocean and resulting climate impacts would be practically irreversible.

### Economic Implications: Need for a Carbon Fee

The implication is that the world must move rapidly to carbon-free energies and energy efficiency, leaving most remaining fossil fuels in the ground, if climate is to be kept close to the Holocene range and climate disasters averted. Is rapid change possible?

The potential for rapid change can be shown by examples. A basic requirement for phasing down fossil fuel emissions is abundant carbon-free electricity, which is the most rapidly growing form of energy and also has the potential to provide energy for transportation and heating of buildings. In one decade (1977–1987), France increased its nuclear power production 15-fold, with the nuclear portion of its electricity increasing from 8% to 70% [231]. In one decade (2001–2011) Germany increased the non-hydroelectric renewable energy portion of its electricity from 4% to 19%, with fossil fuels decreasing from 63% to 61% (hydroelectric decreased from 4% to 3% and nuclear power decreased from 29% to 18%) [231].

Given the huge task of replacing fossil fuels, contributions are surely required from energy efficiency, renewable energies, and nuclear power, with the mix depending on local preferences. Renewable energy and nuclear power have been limited in part by technical challenges. Nuclear power faces persistent concerns about safety, nuclear waste, and potential weapons proliferation, despite past contributions to mortality prevention and climate change mitigation [232]. Most renewable energies tap diffuse intermittent sources often at a distance from the user population, thus requiring large-scale energy storage and transport. Developing technologies can ameliorate these issues, as discussed below. However, apparent cost is the constraint that prevents nuclear and renewable energies from fully supplanting fossil fuel electricity generation.

Transition to a post-fossil fuel world of clean energies will not occur as long as fossil fuels appear to the investor and consumer to be the cheapest energy. Fossil fuels are cheap only because they do not pay their costs to society and receive large direct and indirect subsidies [233]. Air and water pollution from fossil fuel extraction and use have high costs in human health, food production, and natural ecosystems, killing more than 1,000,000 people per year and affecting the health of billions of people [232], [234], with costs borne by the public. Costs of climate change and ocean acidification, already substantial and expected to grow considerably [26], [235], also are borne by the public, especially by young people and future generations.

Thus the essential underlying policy, albeit not sufficient, is for emissions of CO_2_ to come with a price that allows these costs to be internalized within the economics of energy use. Because so much energy is used through expensive capital stock, the price should rise in a predictable way to enable people and businesses to efficiently adjust lifestyles and investments to minimize costs. Reasons for preference of a carbon fee or tax over cap-and-trade include the former’s simplicity and relative ease of becoming global [236]. A near-global carbon tax might be achieved, e.g., via a bi-lateral agreement between China and the United States, the greatest emitters, with a border duty imposed on products from nations without a carbon tax, which would provide a strong incentive for other nations to impose an equivalent carbon tax. The suggestion of a carbon fee collected from fossil fuel companies with all revenues distributed to the public on a per capita basis [237] has received at least limited support [238].

Economic analyses indicate that a carbon price fully incorporating environmental and climate damage would be high [239]. The cost of climate change is uncertain to a factor of 10 or more and could be as high as ∼$1000/tCO_2_ [235], [240]. While the imposition of such a high price on carbon emissions is outside the realm of short-term political feasibility, a price of that magnitude is not required to engender a large change in emissions trajectory.

An economic analysis indicates that a tax beginning at $15/tCO_2_ and rising $10/tCO_2_ each year would reduce emissions in the U.S. by 30% within 10 years [241]. Such a reduction is more than 10 times as great as the carbon content of tar sands oil carried by the proposed Keystone XL pipeline (830,000 barrels/day) [242]. Reduced oil demand would be nearly six times the pipeline capacity [241], thus the carbon fee is far more effective than the proposed pipeline.

A rising carbon fee is the *sine qua non* for fossil fuel phase out, but not enough by itself. Investment is needed in RD&D (research, development and demonstration) to help renewable energies and nuclear power overcome obstacles limiting their contributions. Intermittency of solar and wind power can be alleviated with advances in energy storage, low-loss smart electric grids, and electrical vehicles interacting with the grid. Most of today’s nuclear power plants have half-century-old technology with light-water reactors [243] utilizing less than 1% of the energy in the nuclear fuel and leaving unused fuel as long-lived nuclear “waste” requiring sequestration for millennia. Modern light-water reactors can employ convective cooling to eliminate the need for external cooling in the event of an anomaly such as an earthquake. However, the long-term future of nuclear power will employ “fast” reactors, which utilize ∼99% of the nuclear fuel and can “burn” nuclear waste and excess weapons material [243]. It should be possible to reduce the cost of nuclear power via modular standard reactor design, but governments need to provide a regulatory environment that supports timely construction of approved designs. RD&D on carbon capture and storage (CCS) technology is needed, especially given our conclusion that the current atmospheric CO_2_ level is already in the dangerous zone, but continuing issues with CCS technology [7], [244] make it inappropriate to construct fossil fuel power plants with a promise of future retrofit for carbon capture. Governments should support energy planning for housing and transportation, energy and carbon efficiency requirements for buildings, vehicles and other manufactured products, and climate mitigation and adaptation in undeveloped countries.

Economic efficiency would be improved by a rising carbon fee. Energy efficiency and alternative low-carbon and no-carbon energies should be allowed to compete on an equal footing, without subsidies, and the public and business community should be made aware that the fee will continually rise. The fee for unconventional fossil fuels, such as oil from tar sands and gas from hydrofracking, should include carbon released in mining and refining processes, e.g., methane leakage in hydrofracking [245]–[249]. If the carbon fee rises continually and predictably, the resulting energy transformations should generate many jobs, a welcome benefit for nations still suffering from long-standing economic recession. Economic modeling shows that about 60% of the public, especially low-income people, would receive more money via a per capita 100% dispersal of the collected fee than they would pay because of increased prices [241].

### Fairness: Intergenerational Justice and Human Rights

Relevant fundamentals of climate science are clear. The physical climate system has great inertia, which is due especially to the thermal inertia of the ocean, the time required for ice sheets to respond to global warming, and the longevity of fossil fuel CO_2_ in the surface carbon reservoirs (atmosphere, ocean, and biosphere). This inertia implies that there is additional climate change “in the pipeline” even without further change of atmospheric composition. Climate system inertia also means that, if large-scale climate change is allowed to occur, it will be exceedingly long-lived, lasting for many centuries.

One implication is the likelihood of intergenerational effects, with young people and future generations inheriting a situation in which grave consequences are assured, practically out of their control, but not of their doing. The possibility of such intergenerational injustice is not remote – it is at our doorstep now. We have a planetary climate crisis that requires urgent change to our energy and carbon pathway to avoid dangerous consequences for young people and other life on Earth.

Yet governments and industry are rushing into expanded use of fossil fuels, including unconventional fossil fuels such as tar sands, tar shale, shale gas extracted by hydrofracking, and methane hydrates. How can this course be unfolding despite knowledge of climate consequences and evidence that a rising carbon price would be economically efficient and reduce demand for fossil fuels? A case has been made that the absence of effective governmental leadership is related to the effect of special interests on policy, as well as to public relations efforts by organizations that profit from the public’s addiction to fossil fuels [237], [250].

The judicial branch of governments may be less subject to pressures from special financial interests than the executive and legislative branches, and the courts are expected to protect the rights of all people, including the less powerful. The concept that the atmosphere is a public trust [251], that today’s adults must deliver to their children and future generations an atmosphere as beneficial as the one they received, is the basis for a lawsuit [252] in which it is argued that the U.S. government is obligated to protect the atmosphere from harmful greenhouse gases.

Independent of this specific lawsuit, we suggest that intergenerational justice in this matter derives from fundamental rights of equality and justice. The Universal Declaration of Human Rights [253] declares “All are equal before the law and are entitled without any discrimination to equal protection of the law.” Further, to consider a specific example, the United States Constitution provides all citizens “equal protection of the laws” and states that no person can be deprived of “life, liberty or property without due process of law”. These fundamental rights are a basis for young people to expect fairness and justice in a matter as essential as the condition of the planet they will inhabit. We do not prescribe the legal arguments by which these rights can be achieved, but we maintain that failure of governments to effectively address climate change infringes on fundamental rights of young people.

Ultimately, however, human-made climate change is more a matter of morality than a legal issue. Broad public support is probably needed to achieve the changes needed to phase out fossil fuel emissions. As with the issue of slavery and civil rights, public recognition of the moral dimensions of human-made climate change may be needed to stir the public’s conscience to the point of action.

A scenario is conceivable in which growing evidence of climate change and recognition of implications for young people lead to massive public support for action. Influential industry leaders, aware of the moral issue, may join the campaign to phase out emissions, with more business leaders becoming supportive as they recognize the merits of a rising price on carbon. Given the relative ease with which a flat carbon price can be made international [236], a rapid global emissions phasedown is feasible. As fossil fuels are made to pay their costs to society, energy efficiency and clean energies may reach tipping points and begin to be rapidly adopted.

Our analysis shows that a set of actions exists with a good chance of averting “dangerous” climate change, if the actions begin now. However, we also show that time is running out. Unless a human “tipping point” is reached soon, with implementation of effective policy actions, large irreversible climate changes will become unavoidable. Our parent’s generation did not know that their energy use would harm future generations and other life on the planet. If we do not change our course, we can only pretend that we did not know.

## Discussion

We conclude that an appropriate target is to keep global temperature within or close to the temperature range in the Holocene, the interglacial period in which civilization developed. With warming of 0.8°C in the past century, Earth is just emerging from that range, implying that we need to restore the planet’s energy balance and curb further warming. A limit of approximately 500 GtC on cumulative fossil fuel emissions, accompanied by a net storage of 100 GtC in the biosphere and soil, could keep global temperature close to the Holocene range, assuming that the net future forcing change from other factors is small. The longevity of global warming (Fig. 9) and the implausibility of removing the warming if it is once allowed to penetrate the deep ocean emphasize the urgency of slowing emissions so as to stay close to the 500 GtC target.

Fossil fuel emissions of 1000 GtC, sometimes associated with a 2°C global warming target, would be expected to cause large climate change with disastrous consequences. The eventual warming from 1000 GtC fossil fuel emissions likely would reach well over 2°C, for several reasons. With such emissions and temperature tendency, other trace greenhouse gases including methane and nitrous oxide would be expected to increase, adding to the effect of CO_2_. The global warming and shifting climate zones would make it less likely that a substantial increase in forest and soil carbon could be achieved. Paleoclimate data indicate that slow feedbacks would substantially amplify the 2°C global warming. It is clear that pushing global climate far outside the Holocene range is inherently dangerous and foolhardy.

The fifth IPCC assessment Summary for Policymakers [14] concludes that to achieve a 50% chance of keeping global warming below 2°C equivalent CO_2_ emissions should not exceed 1210 GtC, and after accounting for non-CO_2_ climate forcings this limit on CO_2_ emissions becomes 840 GtC. The existing drafts of the fifth IPCC assessment are not yet approved for comparison and citation, but the IPCC assessment is consistent with studies of Meinshausen et al. [254] and Allen et al. [13], hereafter M2009 and A2009, with which we can make comparisons. We will also compare our conclusions with those of McKibben [255]. M2009 and A2009 appear together in the same journal with the two lead authors on each paper being co-authors on the other paper. McKibben [255], published in a popular magazine, uses quantitative results of M2009 to conclude that most remaining fossil fuel reserves must be left in the ground, if global warming this century is to be kept below 2°C. McKibben [255] has been very successful in drawing public attention to the urgency of rapidly phasing down fossil fuel emissions.

M2009 use a simplified carbon cycle and climate model to make a large ensemble of simulations in which principal uncertainties in the carbon cycle, radiative forcings, and climate response are allowed to vary, thus yielding a probability distribution for global warming as a function of time throughout the 21st century. M2009 use this distribution to infer a limit on total (fossil fuel+net land use) carbon emissions in the period 2000–2049 if global warming in the 21st century is to be kept below 2°C at some specified probability. For example, they conclude that the limit on total 2000–2049 carbon emissions is 1440 GtCO_2_ (393 GtC) to achieve a 50% chance that 21st century global warming will not exceed 2°C.

A2009 also use a large ensemble of model runs, varying uncertain parameters, and conclude that total (fossil fuel+net land use) carbon emissions of 1000 GtC would most likely yield a peak CO_2_-induced warming of 2°C, with 90% confidence that the peak warming would be in the range 1.3–3.9°C. They note that their results are consistent with those of M2009, as the A2009 scenarios that yield 2°C warming have 400–500 GtC emissions during 2000–2049; M2009 find 393 GtC emissions for 2°C warming, but M2009 included a net warming effect of non-CO_2_ forcings, while A2009 neglected non-CO_2_ forcings.

McKibben [255] uses results of M2009 to infer allowable fossil fuel emissions up to 2050 if there is to be an 80% chance that maximum warming in the 21st century will not exceed 2°C above the pre-industrial level. M2009 conclude that staying under this 2°C limit with 80% probability requires that 2000–2049 emissions must be limited to 656 GtCO_2_ (179 GtC) for 2007–2049. McKibben [255] used this M2009 result to determine a remaining carbon budget (at a time not specified exactly) of 565 GtCO_2_ (154 GtC) if warming is to stay under 2°C. Let us update this analysis to the present: fossil fuel emissions in 2007–2012 were 51 GtC [5], so, assuming no net emissions from land use in these few years, the M2009 study implies that the remaining budget at the beginning of 2013 was 128 GtC.

Thus, coincidentally, the McKibben [255] approach via M2009 yields almost exactly the same remaining carbon budget (128 GtC) as our analysis (130 GtC). However, our budget is that required to limit warming to about 1°C (there is a temporary maximum during this century at about 1.1–1.2°C, Fig. 9), while McKibben [255] is allowing global warming to reach 2°C, which we have concluded would be a disaster scenario! This apparently vast difference arises from three major factors.

First, we assumed that reforestation and improved agricultural and forestry practices can suck up the net land use carbon of the past. We estimate net land use emissions as 100 GtC, while M2009 have land use emissions almost twice that large (∼180 GtC). We argue elsewhere (see section 14 in Supporting Information of [54]) that the commonly employed net land use estimates [256] are about a factor of two larger than the net land use carbon that is most consistent with observed CO_2_ history. However, we need not resolve that long-standing controversy here. The point is that, to make the M2009 study equivalent to ours, negative land use emissions must be included in the 21st century equal to earlier positive land use emissions.

Second, we have assumed that future net change of non-CO_2_ forcings will be zero, while M2009 have included significant non-CO_2_ forcings. In recent years non-CO_2_ GHGs have provided about 20% of the increase of total GHG climate forcing.

Third, our calculations are for a single fast-feedback equilibrium climate sensitivity, 3°C for doubled CO_2_, which we infer from paleoclimate data. M2009 use a range of climate sensitivities to compute a probability distribution function for expected warming, and then McKibben [255] selects the carbon emission limit that keeps 80% of the probability distribution below 2°C.

The third factor is a matter of methodology, but one to be borne in mind. Regarding the first two factors, it may be argued that our scenario is optimistic. That is true, but both goals, extracting 100 GtC from the atmosphere via improved forestry and agricultural practices (with possibly some assistance from CCS technology) and limiting additional net change of non-CO_2_ forcings to zero, are feasible and probably much easier than the principal task of limiting additional fossil fuel emissions to 130 GtC.

We noted above that reforestation and improving agricultural and forestry practices that store more carbon in the soil make sense for other reasons. Also that task is made easier by the excess CO_2_ in the air today, which causes vegetation to take up CO_2_ more efficiently. Indeed, this may be the reason that net land use emissions seem to be less than is often assumed.

As for the non-CO_2_ forcings, it is noteworthy that greenhouse gases controlled by the Montreal Protocol are now decreasing, and recent agreement has been achieved to use the Montreal Protocol to phase out production of some additional greenhouse gases even though those gases do not affect the ozone layer. The most important non-CO_2_ forcing is methane, whose increases in turn cause tropospheric ozone and stratospheric water vapor to increase. Fossil fuel use is probably the largest source of methane [1], so if fossil fuel use begins to be phased down, there is good basis to anticipate that all three of these greenhouse gases could decrease, because of the approximate 10-year lifetime of methane.

As for fossil fuel CO_2_ emissions, considering the large, long-lived fossil fuel infrastructure in place, the science is telling us that policy should be set to reduce emissions as rapidly as possible. The most fundamental implication is the need for an across-the-board rising fee on fossil fuel emissions in order to allow true free market competition from non-fossil energy sources. We note that biospheric storage should not be allowed to offset further fossil fuel emissions. Most fossil fuel carbon will remain in the climate system more than 100,000 years, so it is essential to limit the emission of fossil fuel carbon. It will be necessary to have incentives to restore biospheric carbon, but these must be accompanied by decreased fossil fuel emissions.

A crucial point to note is that the three tasks [limiting fossil fuel CO_2_ emissions, limiting (and reversing) land use emissions, limiting (and reversing) growth of non-CO_2_ forcings] are interactive and reinforcing. In mathematical terms, the problem is non-linear. As one of these climate forcings increases, it increases the others. The good news is that, as one of them decreases, it tends to decrease the others. In order to bestow upon future generations a planet like the one we received, we need to win on all three counts, and by far the most important is rapid phasedown of fossil fuel emissions.

It is distressing that, despite the clarity and imminence of the danger of continued high fossil fuel emissions, governments continue to allow and even encourage pursuit of ever more fossil fuels. Recognition of this reality and perceptions of what is “politically feasible” may partially account for acceptance of targets for global warming and carbon emissions that are well into the range of “dangerous human-made interference” with climate. Although there is merit in simply chronicling what is happening, there is still opportunity for humanity to exercise free will. Thus our objective is to define what the science indicates is needed, not to assess political feasibility. Further, it is not obvious to us that there are physical or economic limitations that prohibit fossil fuel emission targets far lower than 1000 GtC, even targets closer to 500 GtC. Indeed, we suggest that rapid transition off fossil fuels would have numerous near-term and long-term social benefits, including improved human health and outstanding potential for job creation.

A world summit on climate change will be held at United Nations Headquarters in September 2014 as a preliminary to negotiation of a new climate treaty in Paris in late 2015. If this treaty is analogous to the 1997 Kyoto Protocol [257], based on national targets for emission reductions and cap-and-trade-with-offsets emissions trading mechanisms, climate deterioration and gross intergenerational injustice will be practically guaranteed. The palpable danger that such an approach is conceivable is suggested by examination of proposed climate policies of even the most forward-looking of nations. Norway, which along with the other Scandinavian countries has been among the most ambitious and successful of all nations in reducing its emissions, nevertheless approves expanded oil drilling in the Arctic and development of tar sands as a majority owner of Statoil [258]–[259]. Emissions foreseen by the Energy Perspectives of Statoil [259], if they occur, would approach or exceed 1000 GtC and cause dramatic climate change that would run out of control of future generations. If, in contrast, leading nations agree in 2015 to have internal rising fees on carbon with border duties on products from nations without a carbon fee, a foundation would be established for phaseover to carbon free energies and stable climate.

## Supporting Information

Table S1 (ODS)

Table S2 (ODS)

Table S3 (ODS)

Text S1 (DOC)
