# Supplementary material for: Assessing “Dangerous Climate Change”: Required Reduction of Carbon Emissions to Protect Young People, Future Generations and Nature
Source: PLoS One. 2013 Dec 3;8(12):e81648. doi: 10.1371/journal.pone.0081648 (PMC3849278; doi:10.1371/journal.pone.0081648)
Supplement: Text S1 — (DOC) [file pone.0081648.s004.doc]

**Supporting Information**

**1. Paleocene-Eocene Thermal Maximum (PETM)**

Rapid global warming of at least 5 °C at the Paleocene-Eocene boundary (about 56 million years ago) provides valuable insights into the carbon cycle, climate system, and biotic responses to environmental change [S1, S2, S3]. Although the PETM is far from a perfect analog to the potential climate change from humans burning of fossil fuels, it did involve the injection of a likely 4000-7000 Gt of carbon into the atmosphere and ocean [S3], which is comparable to the potential injection from burning most fossil fuels. On the other hand, the PETM warming occurred over 5-10 thousand years [S3, S4, S5], while the fossil fuel injection is likely to be much more rapid. Moreover, the onset of the PETM was accompanied by intensification of the hydrologic cycle, with some regions (mid- to high latitudes) becoming much wetter, other regions dryer [S6, S7]. In regions that became drier, the delivery of precipitation appears to have become more seasonally distributed (monsoon-like). High-energy flooding events increased in frequency and intensity.

The most common interpretation of the PETM is that the carbon originated mainly from melting of methane hydrates. A potential alternative carbon source is release from Antarctic permafrost and peat [S8]. A question of whether the methane hydrate source could be large enough, given the warmer ocean at that time, has been addressed with an affirmative conclusion [S9]. Regardless of the carbon source, PETM occurred during a period of slow long-term global warming that was presumably driven by increasing volcanic carbon emissions associated with plate tectonics [S10], which suggests that methane release may have been initiated at a physical threshold, acting as a powerful feedback magnifying that warming. Support for this interpretation that the carbon release was an amplifying feedback is provided by evidence that several other PETM-like events in Earth's history (spikes in global warming and light-carbon sediments) were astronomically paced, i.e., they occurred during the warming phase of climate oscillations associated with perturbations of Earth's orbit [S1].

The PETM witnessed global scale disruption of marine and terrestrial ecosystems with mass migration, temporary redistribution of many species toward higher latitude, and rapid evolution, particularly toward dwarfism of mammals, but with only minor extinctions [S3]. The evolution toward smaller body size may have been a result of a decline in biological productivity and food availability [S11].

An important point is that the magnitude of the PETM carbon injection and warming is comparable to what will occur if humanity burns most of the fossil fuels, but the human-made warming is occurring 10-100 times faster. We have no empirical evidence on the ability of life on Earth to maintain itself during such a large, rapid climate change, with climate zones shifting much faster than species have ever experienced. The faster carbon addition also means that acidification and carbonate dissolution in the surface ocean would be more severe than that experienced by surface-dwelling organisms in the PETM.

**2. Human Health**

If fossil fuel emissions continue to increase rapidly substantial impacts of climate change on human health are likely. Some effects are already beginning to occur.

**Infectious Disease.** Higher temperature and flooding affect water quality and facilitate spread of infectious diseases by increasing the range and frequency of conditions favoring blood-sucking arthropods, such as mosquitoes, fleas, lice, biting flies, bugs and ticks [S12]. These climate effects exacerbate already high disease and death rates in developing countries [S13]. Warming is causing traditionally tropical diseases to become more prevalent at higher latitudes, including Europe [S14] and the United States [S15]. Warmer winters and polar amplification of warming increase the range of these disease-bearing vectors.

**Crop Pests and Disease.** Warming fortifies pests and weakens hosts in forests, agricultural systems, and marine life. Warmer winters allow pine bark beetles to overwinter and expand their range, to the detriment of boreal forests [S16]. Climate trends also favor expansion of the Asian long-horned beetle and wooly adelgid to the detriment of trees in the Northeast United States [S17]. Warming increases the range of pests such as white flies, aphids and locust that damage crops, and it stimulates growth of agricultural weeds, leading to increased use of pesticides and herbicides that themselves are harmful to human health. Warming harms coral and other species hosted by coral reefs, and, along with excess nutrients from fertilizers, contributes to harmful algal blooms that cause dead zones in coastal waterways and estuaries [S18].

**Heat Waves and Droughts.** Global warming, although "only" 0.8 °C in the past century, is already sufficient to substantially increase the likelihood of extreme heat waves and droughts. The probability of occurrence of extreme anomalies as great as the Moscow heat wave in 2010 and the Texas/Oklahoma heat wave and drought of 2011 has increased by several times because of global warming [S18], and the probability will increase even further if global warming continues to increase. Heat waves cause illness and death and also can lead to an increase in aggression, including violent assaults [S19] and suicide [S20].

**Food Insecurity.** Food supplies are compromised by increasing climate extremes, crop pests, and displacement of food crops by biofuel crops [S21]. Food shortages and price hikes contribute to malnutrition and poor health that increase vulnerability to infectious diseases, and also are frequently factors in conflicts and wars [S21].

**REFERENCES**

[S1] Zachos J, Pagani M, Sloan L, Thomas E, Billups K (2001) Trends, rhythms, and

aberrations in global climate 65 Ma to present. Science 292: 686-693.

[S2] Kennett JP, Stott LD (1991) Abrupt deep sea warming, paleoceanographic changes, and benthic extinctions at the end of the Palaeocene. Nature 353: 319-322.

[S3] Dunkley Jones T, Ridgwell A, Lunt DJ, Maslin MA, Schmidt DN, Valdes PJ (2010) A Palaeogene perspective on climate sensitivity and methane hydrate instability. Phil Trans Roy Soc A 368: 2395-2415.

[S4] Zeebe RE, Zachos JC, Dickens GR (2009) Carbon dioxide forcing alone insufficient to explain Palaeocene-Eocene Thermal Maximum warming. Nat Geosci 2: 576-580.

[S5] Cui Y, Kump LR, Ridgwell AJ, Charles AJ, Junium CK, et al. (2011) Slow release of fossil carbon during the Palaeocene–Eocene Thermal Maximum. Nat Geosci 4: 481-485.

[S6] Schmitz B, Pujalte V (2007) Abrupt increase in seasonal extreme precipitattion at the Paleocene-Eocene boundary. Geology 35, 215-218.

[S7] Foreman BZ, Heller PL, Clementz MT (2012) Fluvial response to abrupt global warming at the Palaeocene/Eocene boundary. Nature 491, 92-95.

[S8] DeConto RM, Galeotti S, Pagani M, Tracy D, Schaefer K, et al. (2012) Past extreme wrming events linked to massive carbon release from thawing permafrost. Nature 484, 87-91.

[S9] Gu G, Dickens GR, Bhatnagar G, Colwell FS, Hirasaki GJ, Chapman WG (2011) Abundant Early Palaeogene marine gas hydrates despite warm deep-ocean temperatures. Nat Geosci 4: 848-851.

[S10] Kent DV, Muttoni G (2008) Equatorial convergence of India and early Cenozoic climate trends. Proc Natl Acad Sci USA 105: 16065-16070.

[S11] Chester S, Bloch J, Secord R, Boyer D (2010) A new small-bodied species of Palaeonictis (Creodonta, Oxyaenidae) from the Paleocene-Eocene Thermal Maximum. J Mamm Evol 17: 227-243.

[S12] Patz JA, Campbell-Lendrum D, Holloway T, Foley JA (2005) Impact of regional climate change on human health. Nature 438: 310-317.

[S13] Ashbolt NJ (2004) Microbial contamination of drinking water and disease outcomes in developing regions. Toxicology 198: 229-238.

[S14] Semenza JC, Menne B (2009) Climate change and infectious diseasees in Europe. Lancet 9: 365-375.

[S15] Patz JA, McGeehin MA, Bernard SM, Ebi KL, Epstein PR, et al. (2000) The potential health impacts of climate variability and change for the United States. Environ Health Perspec 108: 367-376.

[S16] Raffa KF, Aukema BH, Bentz BJ, Carroll AL, Hicke JA, et al. (2008) Cross-scale drivers of natural disturbances prone to anthropogenic amplification: the dynamics of bark beetle eruptions. Biosci 58: 501-517.

[S17] Ayres MP, Lombardero MJ (2000) Assessing the consequences of global change for forest disturbance from herbivores and pathogenss. Sci Total Environ 262: 263-286.

[S18] Diaz RJ, Rosenberg R (2008) Spreading dead zones and consequences for marine ecosystems. Science 321: 926-928.

[S19] Bushman BJ, Wang MC, Anderson CA (2005) Is the Curve Relating Temperature to Aggression Linear or Curvilinear? Assaults and Temperature in Minneapolis Reexamined. J Personality Social Psychology 89: 62-66.

[S20] Page LA, Hajat S, Kovats RS (2007) Relationship between daily suicide counts and temperature in England and Wales. British J Psych 191: 106-112.

[S21] Intergovernmental Panel on Climate Change (2007) Climate Change 2007: Mitigation of

Climate Change. Metz B, Davidson OR, Bosch PR, Dave R, Meyer LA, editors.

Cambridge: Cambridge University Press.
